# Chlorination of epithelial tight junction proteins by neutrophil myeloperoxidase promotes barrier dysfunction and mucosal inflammation

**DOI:** 10.1172/jci.insight.178525

**Published:** 2024-06-11

**Authors:** Ian M. Cartwright, Liheng Zhou, Samuel D. Koch, Nichole Welch, Daniel Zakharov, Rosemary Callahan, Calen A. Steiner, Mark E. Gerich, Joseph C. Onyiah, Sean P. Colgan

**Affiliations:** 1 Mucosal Inflammation Program and; 2 Department of Medicine, University of Colorado Anschutz Medical Campus, Aurora, Colorado, USA.; 3 Rocky Mountain Regional Veterans Affairs Medical Center, Aurora, Colorado, USA.; 4 School of Medicine, University College Dublin, Dublin, United Kingdom.

**Keywords:** Inflammation, Inflammatory bowel disease, Neutrophils

## Abstract

Neutrophils (polymorphonuclear leukocytes, PMNs) comprise a major component of the immune cell infiltrate during acute mucosal inflammation and have an important role in molding the inflammatory tissue environment. While PMNs are essential to clearance of invading microbes, the major PMN antimicrobial enzyme myeloperoxidase (MPO) can also promote bystander tissue damage. We hypothesized that blocking MPO would attenuate acute colitis and prevent the development of chronic colitis by limiting bystander tissue damage. Using the acute and chronic dextran sodium sulfate model of murine colitis, we demonstrated that MPO-deficient mice experienced less inflammation and more rapidly resolved colitis relative to wild-type controls. Mechanistic studies demonstrated that activated MPO disrupted intestinal epithelial barrier function through the dysregulation of the epithelial tight junction proteins. Our findings revealed that activated MPO chlorinates tyrosine within several tight junction proteins, thereby promoting tight junction mislocalization and dysfunction. These observations in cell models and in murine colitis were validated in human intestinal biopsies from individuals with ulcerative colitis and revealed a strong correlation between disease severity (Mayo score) and tissue chlorinated tyrosine levels. In summary, these findings implicate MPO as a viable therapeutic target to limit bystander tissue damage and preserve mucosal barrier function during inflammation.

## Abstract 2

Myloperoxidase-deficient mice develop less inflammation in both acute and chronic models of colitis due to decreased inflammatory cytokines and increased resolution.

## Introduction

A hallmark of mucosal inflammatory disease is the accumulation of neutrophils (PMNs) at sites of injury. PMNs are central components of the innate immune system and are essential for clearance of microbes at sites of inflammation. It is notable, however, that in diseases such as ulcerative colitis (UC) and Crohn’s disease, PMNs are not efficiently cleared from the tissue and may contribute to bystander tissue damage (1). This bystander tissue damage is often attributed to the PMN antimicrobial functions. The primary PMN antimicrobial enzyme, myeloperoxidase (MPO), composes approximately 5% of cell dry weight (2). Within the PMN phagosome, MPO is responsible for the generation of hypochlorous acid (HOCl) from hydrogen peroxide and halides such as chlorine (3, 4). HOCl is a potent 2-electron oxidant that diffuses through the tissue and indiscriminately reacts with sulfur and nitrogen atoms. Specifically, HOCl reacts with the phenol moiety of tyrosine (Tyr) and generates the chlorinated product 3-chloro-tyrosine (3-Cl-Tyr). 3-Cl-Tyr is heat stable and has been demonstrated to be a reliable marker of HOCl oxidation and an in vivo marker of MPO activity (3, 5–7).

MPO is not confined to the phagosome; PMNs also secrete MPO into the extracellular space during degranulation (8). MPO itself can induce additional degranulation of PMNs, furthering its liberation into the tissue microenvironment and thereby creating a positive feedback loop (9, 10). It is well established that PMNs are required for intestinal homeostasis and are essential for maintaining epithelial barrier function and prompting resolution during the inflammatory response (11, 12). Despite these beneficial aspects of the PMN, uncontrolled PMN activity promotes bystander tissue damage and can attenuate the resolution of inflammation (13–15). Recently, it was demonstrated that targeting MPO has the potential to limit pathogenic PMN activity. For example, inhibition of MPO by both synthetic inhibitors and microbiota-derived indoles have been shown to improve acute dextran sodium sulfate–induced (DSS-induced) colitis (5, 16, 17). Additionally, it has been reported that PMN-derived extracellular vesicles, containing MPO, inhibit wound healing both in vitro and in vivo (18). However, it is not well understood how inhibition of MPO affects the resolution of acute inflammation or the development of chronic inflammation.

Despite the propensity for PMNs to cause tissue damage, PMNs have been shown to have an essential role in molding the inflammatory microenvironment and promoting resolution of inflammation. For example, PMN transepithelial migration depletes local O_2_, which stabilizes hypoxia-inducible factor and triggers the induction of pro-resolution and wound healing pathways (12, 19, 20). Additionally, PMN infiltration is associated with the release of a large number of signaling nucleotides that also induce several antiinflammatory and wound healing pathways (12, 21). Targeting potentially pro-inflammatory pathways in PMNs while preserving their pro-resolution functions may diminish disease severity and prevent progression to chronicity. The current study aims to examine the impact of PMN MPO on the development of chronic colitis in an experimental mouse model of colitis elicited by repeated induction of DSS colitis.

## Results

### Loss of MPO attenuates acute DSS colitis severity.

**Figure 1:**
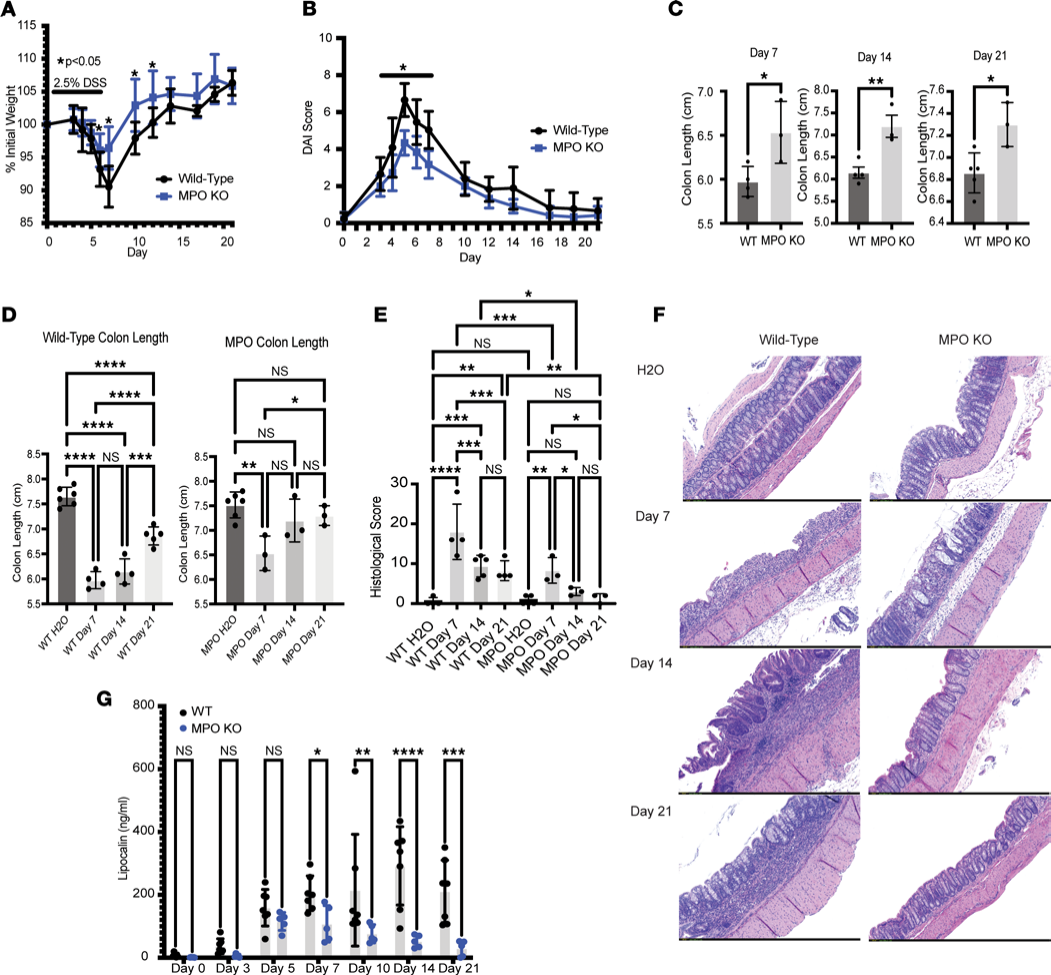
MPO promotes intestinal inflammation in a DSS model of murine colitis. (**A**) Percentage weight loss and (**B**) disease activity index (DAI) from WT and MPO-KO mice that received 2.5% DSS in their drinking water for 5 days and were followed for an additional 16 days, for a total of 21 days. (**C** and **D**) Colon lengths from mice treated with 2.5% DSS in drinking water for 5 days collected at days 7, 14, and 21. (**E**) Histological score from distal colon tissue harvested at days 7, 14, and 21. (**F**) Representative microscopy (original magnification, ×10) images of hematoxylin and eosin–stained colon tissue at days 7, 14, and 21 of the DSS experiment. (**G**) Analysis of fecal lipocalin from fecal pellets collected at days 0, 3, 5, 7, 10, 14, and 21; mice were treated with 2.5% DSS for 5 days. (**A**–**F**) *n* = 3–5 and (**G**) *n* = 5–7 mice per group. Data are expressed as mean ± SD, and the *P* value was determined by *t* test (**C**), 1-way ANOVA (**D**, **E**, and **G**), or 2-way ANOVA (**A** and **B**) where appropriate. *P < 0.05, **P < 0.01, ***P < 0.001, ****P < 0.0001.

As an initial series of experiments to define the contribution of MPO to colitis, we examined the severity and resolution of acute colitis in wild-type (WT) and MPO-deficient (MPO-KO) mice. After 5 days on DSS (2.5% in drinking water), mice were allowed to recover for 2, 12, or 19 days. As shown in Figure 1A, starting on day 6, the MPO-KO mice showed significantly less weight loss when compared with WT mice (*P* < 0.02). On day 7, when peak weight loss is typically observed in the DSS-induced colitis model, the WT mice had on average approximately 10% weight loss compared with approximately 4% in the MPO-KO mice (*P* < 0.001). MPO-KO mice continued to show significantly less weight loss when compared with WT mice through day 12 (*P* < 0.05). The weight loss was supported by disease activity index (DAI) observations, where MPO-KO mice had significantly lower DAI when compared with WT mice from days 4–7 (Figure 1B). Next, we examined the colon lengths of the MPO-KO and WT mice on days 7, 14, and 21 (2, 12, and 19 days after DSS), with shorter colons correlating with increased disease severity. As shown in Figure 1C, colitic MPO-KO mice had significantly longer colons then the WT mice at days 7, 14, and 21 (*P* < 0.05). When compared with water controls, no significant differences in colon length were observed in MPO-KO mice beyond day 14 (Figure 1D). In WT mice, the colons of the DSS-treated mice were significantly shorter than colons from water controls on days 14 and 21 (Figure 1D, *P* < 0.01). Furthermore, MPO-KO mice had significantly less histological disease at days 7, 14, and 21 when compared with WT mice (Figure 1E, *P* < 0.01). In the MPO-KO mice starting at day 14, there was no significant difference in histological score between the DSS-treated mice and water controls. In the WT mice, there was still significant evidence of histological disease when comparing DSS-treated mice and water controls at day 14 and day 21. Representative histological images from WT and MPO-KO water controls and DSS-treated mice at days 7, 14, 21 are shown in Figure 1F. This analysis revealed substantially increased epithelial denudation, crypt damage, and inflammatory infiltrate in WT mice compared with MPO-KO mice. By days 14 and 21, mucosal wound healing was observed in both WT and MPO-KO mice, while immune cell infiltrate remained high in WT compared with MPO-KO mice. Such observations implicate more rapid and complete inflammatory resolution in the absence of MPO.

### Loss of MPO limits molecular markers of inflammation.

A common diagnostic marker of human intestinal inflammation is fecal calprotectin. In addition to use as a diagnostic marker, fecal calprotectin has been shown to correlate with disease severity (22). In murine models of colitis, fecal lipocalin is a reliable surrogate of calprotectin and a sensitive marker of intestinal inflammation (23). Fecal lipocalin levels were analyzed in the MPO-KO and WT mice throughout DSS treatment. As shown in Figure 1G, fecal lipocalin levels in MPO-KO mice were significantly lower than WT mice starting on day 7 and continuing through day 21. This decrease in lipocalin indicates that the colons in the MPO-KO mice were less inflamed than the WT mice, which supports the observation of longer colon lengths and lower histological scores in the MPO-KO mice (Figure 1, D and E). In addition to the colon lengths and histological scores, tissue cytokines were analyzed by both MESO scale, a multiarray assay for detecting cytokines, and quantitative PCR (qPCR) on day 7. MPO-KO mice expressed significantly less tissue KC/GRO (IL-8 related protein in rodents), TNF-α, IL-6, IFN-γ, and IL-1β compared with WT mice (Supplemental Figure 1, A and B; supplemental material available online with this article; https://doi.org/10.1172/jci.insight.178525DS1). Cytokine levels decreased in both WT and MPO-KO mice between day 7 and day 21; however, there were still significant elevations in tissue KC/GRO, TNF-α, IL-6, and IL-1β observed in the WT mice compared with MPO-KO mice at day 21 (Supplemental Figure 1C). Thus, sensitive biomarkers of inflammation reveal a more rapid inflammatory resolution in the absence of MPO.

### MPO promotes chronic mucosal inflammation.

**Figure 2:**
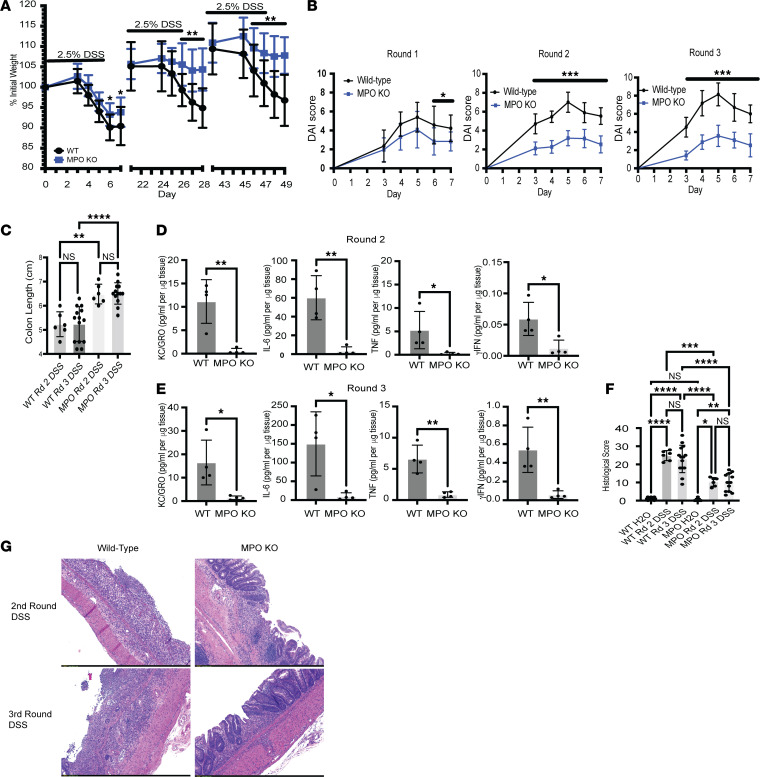
MPO promotes chronic colitis. (**A**) Percentage weight loss and (**B**) DAI from WT and MPO-KO mice that received 2.5% DSS in their drinking water for 5 days and were allowed to recover for 16 days before receiving additional rounds of 2.5% DSS for 5 days. Mice received 1, 2, or 3 rounds of 2.5% DSS. (**C**) Colon lengths from WT and MPO-KO mice collected after 2 or 3 rounds of 2.5% DSS. (**D** and **E**) MESO scale analysis of distal colon tissue collected 2 days after DSS was removed in WT and MPO-KO mice after 2 or 3 rounds of 2.5% DSS. The tissue was analyzed for KC/GRO, IL-6, TNF-α, and IFN-γ. (**F**) Histological score from distal colon tissue harvested at the end of 2 or 3 rounds of 2.5% DSS. (**G**) Representative microscopy (original magnification, ×10) images of hematoxylin and eosin–stained colon tissue after 2 or 3 rounds of 2.5% DSS. *n* = 6–14 mice per group (**A**–**C**, **F**, and **G**). *n* = 4 mice per group (**D** and **E**). Data are expressed as mean ± SD, and the *P* value was determined by *t* test (**D** and **E**), 1-way ANOVA (**C** and **F**), or 2-way ANOVA (**A** and **B**) where appropriate. *P < 0.05, **P < 0.01, ***P < 0.001, ****P < 0.0001.

Given the elevated fecal lipocalin and cytokines at day 21, we extended our studies to interrogate the impact of MPO on chronic DSS colitis. MPO-KO and WT mice were subjected to chronic DSS colitis (3 rounds of DSS separated by 16-day equilibration periods). As shown in Figure 2A, MPO-KO mice lost significantly less weight when compared with WT mice during the second and third rounds of DSS. In addition to a decrease in weight loss, as shown in Figure 2B, MPO-KO mice had significantly lower DAIs during the second and third rounds of DSS when compared with WT mice (~3 compared with ~7, respectively, *P* < 0.001). This progressive decrease in DAI was driven by a significant decrease in bleeding in the MPO-KO cohort during the second and third rounds of DSS (Supplemental Figure 2A). WT mice showed colons that were over 1 cm shorter than the MPO-KO mice after the second and third rounds of DSS (Figure 2C, *P* < 0.01). As for tissue pro-inflammatory cytokines, MPO-KO mice had significantly lower expression of KC/GRO, IL-6, TNF-α, IFN-γ, and IL-1β when compared with WT mice at the end of both the second and third rounds of DSS (Figure 2, D and E, *P* < 0.05). In addition to protein expression of these pro-inflammatory cytokines, we examined transcription expression of these cytokines following the second and third rounds of DSS. MPO-KO mice showed decreased transcriptional expression of KC/GRO, IL-6, TNF-α, IFN-γ, and IL-1β when compared with WT mice at the end of the second and third rounds of DSS (Supplemental Figure 2, B and C, *P* < 0.05). Finally, histological scoring of the tissue supported the prior observations that MPO-KO mice had significantly less chronic inflammation than the WT mice (Figure 2, F and G), with enhanced crypt recovery and decreased immune infiltrate consistent with increased inflammatory resolution in the absence of MPO. It has been previously reported that mice deficient in NADPH oxidase (chronic granulomas disease–like mice), which is an enzyme upstream of MPO, develop spontaneous granulomas in mucosal surfaces (24, 25). Based on these findings we examined the colons of WT and MPO-KO mice from both acute and chronic DSS models for the presence of granulomas. In both the acute and chronic DSS models, the WT mice had significantly more granulomas per centimeter colon than the MPO-KO mice (Supplemental Figure 3A, *P* < 0.005). These results suggest that NADPH oxidase and MPO have distinct roles in the inflammatory process.

### Tissue 3-chloro-tyrosine correlates with disease severity.

**Figure 3:**
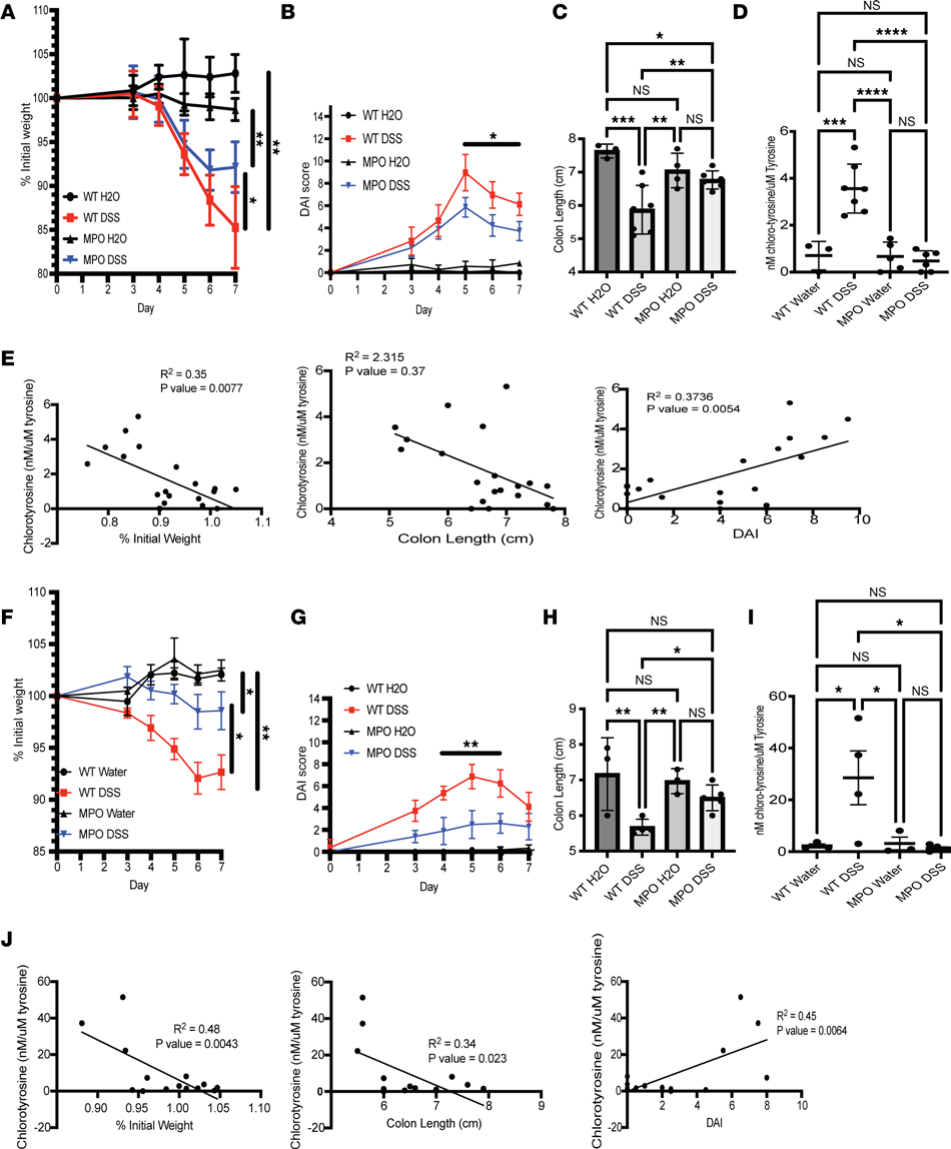
Tissue 3-Cl-Tyr correlates with disease severity. Percentage weight loss from WT and MPO-KO mice following acute (1 round) (**A**) and chronic (2 rounds) (**F**) DSS. Mice were treated with 2.5% DSS for 5 days and collected on day 7 for acute DSS or given a second round of 2.5% DSS at day 21 and collected on day 7 after the second round for chronic DSS. DAI from WT and MPO-KO mice during acute (**B**) and chronic (**G**) DSS. Colon lengths of WT and MPO-KO mice after acute (**C**) and chronic (**H**) DSS. Analysis of 3-Cl-Tyr from colon tissue harvested from WT and MPO-KO mice following acute (**D**) and chronic (**I**) DSS. Pearson correlation between tissue 3-Cl-Tyr and percentage weight loss, colon length, and DAI following acute (**E**) and chronic (**J**) DSS. *n* = 3–7 mice per group. Data are expressed as mean ± SD, and the *P* value was determined by 1-way ANOVA (**C**, **D**, **H**, and **I**) or 2-way ANOVA (**A**, **B**, **F**, and **G**) where appropriate. *P < 0.05, **P < 0.01, ***P < 0.001, ****P < 0.0001.

A specific marker of in vivo MPO activation is the chlorination of the amino acid Tyr (3-chloro-tyrosine, 3-Cl-Tyr) (26). Furthermore, 3-Cl-Tyr is a reliable biomarker for MPO-mediated tissue damage (5–7). To examine the incidence of 3-Cl-Tyr in murine colitis, WT and MPO-KO mice were subjected to 1 (Figure 3, A–E) or 2 (Figure 3, F–J) rounds of DSS. Mice were analyzed for weight loss (Figure 3, A and F), DAI (Figure 3, B and G), colon length (Figure 3, C and H), and magnitude of tissue 3-Cl-Tyr (Figure 3, D and I). We have previously developed an HPLC assay incorporating electrochemical detection and HPLC (EC-HPLC) to distinguish Tyr and 3-Cl-Tyr at low concentrations (ref. 5, and see Supplemental Figure 5). Using this method, we examined levels of 3-Cl-Tyr in tissue from WT and MPO-KO mice after the 1 or 2 rounds of DSS. As shown in Figure 3, D and I, 3-Cl-Tyr was detected at significantly higher levels in WT mice treated with DSS when compared with water controls (*P* < 0.001). This analysis revealed that colitic MPO-KO mice showed no higher 3-Cl-Tyr levels than water controls. Furthermore, there was a roughly 10-fold increase in 3-Cl-Tyr when comparing chronic DSS-treated WT mice with acute DSS-treated WT mice. This increase in 3-Cl-Tyr can be attributed to the increase in histological score and DAI we observed in the WT mice treated with chronic DSS when compared with the acute model. Data from both WT and MPO-KO mice were pooled and examined for correlation between 3-Cl-Tyr levels and weight loss, colon length, and DAI. In both acute and chronic DSS models, there was a negative correlation between 3-Cl-Tyr and both weight loss and colon length. We also observed a positive correlation between 3-Cl-Tyr and DAI (Figure 3, E and J). To ensure that the decrease in 3-Cl-Tyr was not a result of decreased PMN infiltrate, we examined the presence of PMNs in inflamed tissue from WT and MPO-KO mice following 1, 2, or 3 rounds of DSS. At baseline, there was a small difference between WT and MPO-KO mice, with the MPO-KO mice having fewer tissue PMNs compared with WT mice (Supplemental Figure 3B, *P* < 0.05). When examining infiltrating PMNs during active disease, there was no significant difference when comparing WT and MPO-KO mice after each round of DSS (Supplemental Figure 3C).

### MPO inhibitor indole propionic acid protects against chronic inflammation.

**Figure 4:**
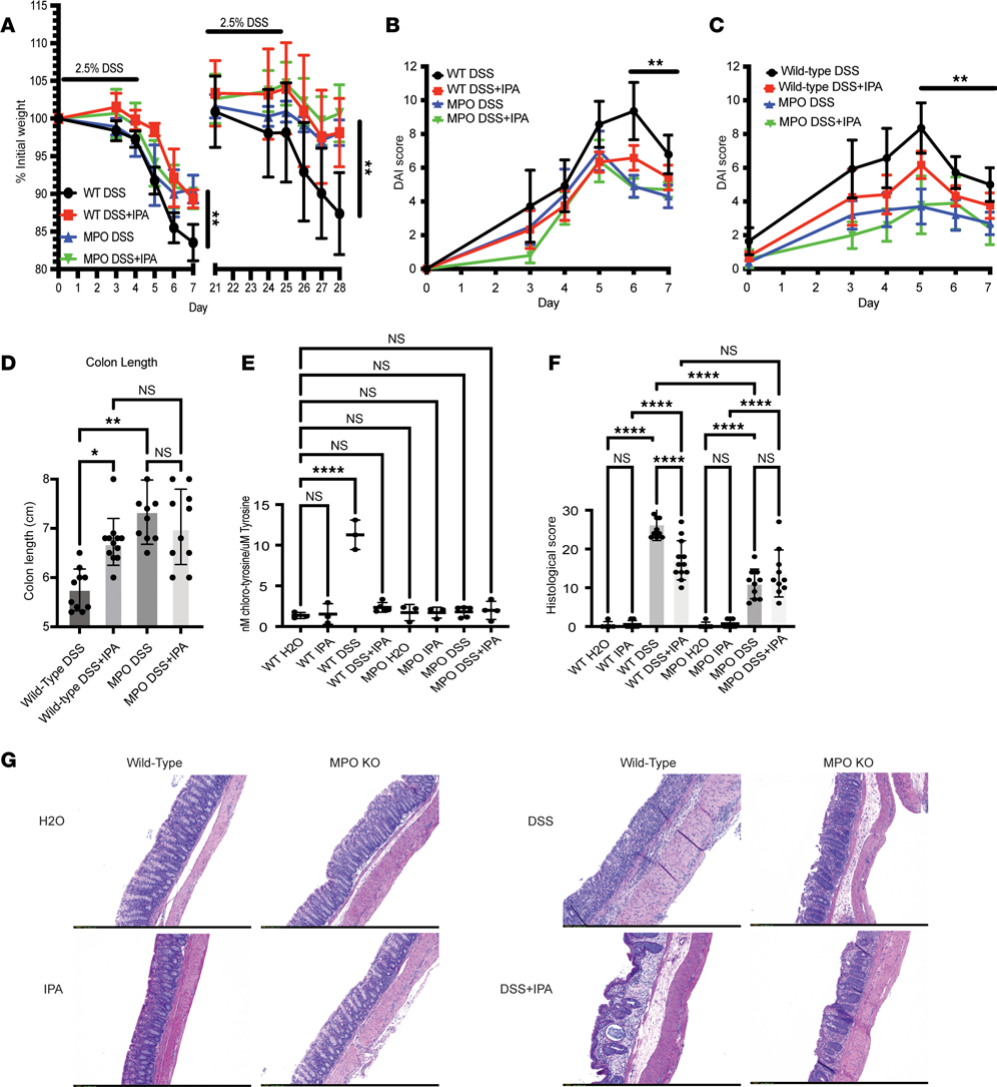
Treatment with IPA protects against chronic DSS. Percentage weight loss from WT and MPO-KO mice during acute (1 round) and chronic (2 rounds) DSS (**A**). Mice were treated with 2.5% DSS for 5 days and collected on day 7 for acute DSS or given a second round of 2.5% DSS at day 21 and collected on day 7 after the second round for chronic DSS. IPA-treated mice were maintained on 0.1 mg/mL IPA in drinking water throughout the duration of the experiment, including between rounds of DSS. DAI from WT and MPO-KO mice treated with IPA during acute (**B**) and chronic (**C**) DSS. Colon length from WT and MPO-KO mice treated with IPA after chronic DSS (**D**). Analysis of 3-Cl-Tyr in WT and MPO-KO mice treated with IPA following chronic DSS (**E**). Histological score from distal colon tissue harvested following chronic DSS colitis (**F**). Representative microscope (original magnification, ×10) images of hematoxylin and eosin–stained colon tissue after chronic DSS colitis 0. *n* = 5–12 mice per group (**A**–**D** and **G**). *n* = 3–5 mice per group (**E** and **F**). Data are expressed as mean ± SD, and the *P* value was determined by 1-way ANOVA (**D**–**F**) or 2-way ANOVA (**A**–**C**) where appropriate. *P < 0.05, **P < 0.01, ****P < 0.0001.

We previously reported that bacterially derived indole molecules inhibit MPO (5). Thus, as a pharmacological approach, we extended our analysis of MPO in colitic mice by examining the impact of indole-3-propionic acid (IPA) on the development of chronic inflammation. As shown in Figure 4A, WT mice treated with 0.1 mg/mL IPA in drinking water and DSS experienced significantly less weight loss when compared with WT treated with DSS only following 1 and 2 rounds of DSS. IPA treatment also decreased DAI when compared with WT controls during both acute and chronic DSS (Figure 4, B and C, respectively). When examining colon length, WT mice treated with IPA had significantly longer colons when compared with untreated WT mice (Figure 4D). To verify MPO inhibition by IPA, we examined levels of 3-Cl-Tyr in inflamed tissue. 3-Cl-Tyr was not elevated in IPA-treated DSS mice compared to water only controls (Figure 4E). When examining the expression of pro-inflammatory signals, we observed a decrease in IL-6, KC/GRO, IFN-γ, and IL-1β in the WT mice treated with IPA when compared with DSS-only WT mice (Supplemental Figure 4). IPA treatment did not appear to decrease TNF-α expression in the WT mice. This elevated expression of TNF-α may be the result of fibrosis in the chronic model of DSS; it has been reported within the liver that IPA treatment is associated with elevated TNF-α expression in liver models of fibrosis (27). MPO-KO mice exposed to the combination of DSS and IPA did not change the expression of pro-inflammatory signals when compared to DSS-only MPO-KO mice. Histological scoring of the inflamed tissue supported the observation that IPA treatment improved chronic colitis in WT mice but had no impact on colitis in the MPO-KO mice (Figure 4, F and G). These results indicate that the protection afforded by genetic loss of MPO can be corroborated with an MPO inhibitor in vivo.

### MPO chlorinates the tight junction protein occludin.

**Figure 5:**
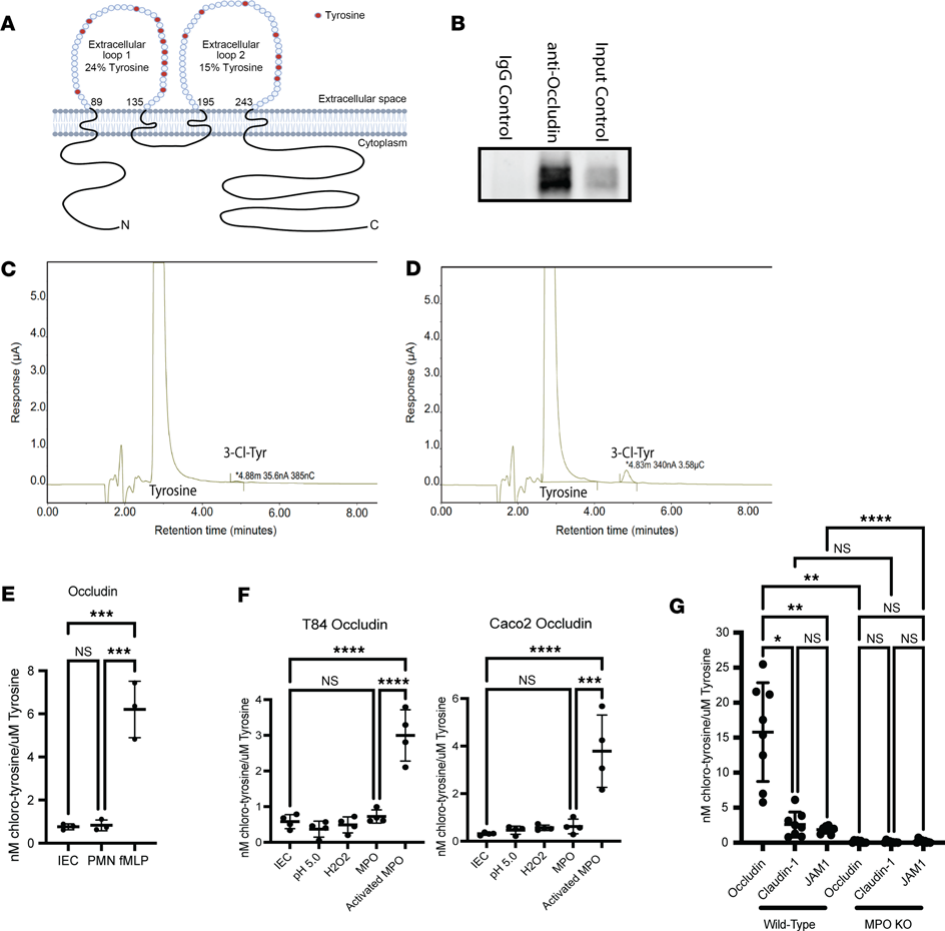
PMN transmigration results in chlorination of occludin. (**A**) Model of the extracellular loops of occludin depicting the location of Tyr residues. (**B**) Western blot of occludin IP. (**C** and **D**) EC-HPLC tracing of Tyr and 3-Cl-Tyr from IP occludin isolated from T84 IECs exposed to nonactivated PMNs (**C**) or activated PMNs (**D**). (**E**) Analysis of the peak area of 3-Cl-Tyr from IP occludin isolated from T84 IECs exposed to inactivated PMNs (PMN) and activated PMNs (fMLP). *n* = 3 biological replicates. Each biological replicate was performed in triplicate. (**F**) Analysis of 3-Cl-Tyr in occludin from T84 and Caco-2 IECs exposed to pH 5.0, hydrogen peroxide, MPO, and activated MPO. *n* = 4 biological replicates. (**G**) Analysis of 3-Cl-Tyr in IP occludin, claudin-1, and JAM-1 isolated from WT and MPO-KO mice treated with 3 rounds of 3% DSS (*n* = 8 WT and 9 MPO-KO). Data are expressed as mean ± SD, and the *P* value was determined by 1-way ANOVA. *P < 0.05, **P < 0.01, ***P < 0.001, ****P < 0.0001.

We next extended our Tyr chlorination results to determine if select tight junction (TJ) proteins might be impacted by MPO. When examining the amino acid sequence of the extracellular domain of the TJ protein occludin, we noted that the 2 extracellular loops of occludin were disproportionately enriched in Tyr residues (24% and 15% Tyr in extracellular loops 1 and 2, respectively, see Figure 5A). Other TJ proteins contain fewer Tyr residues (e.g., extracellular domains of junctional adhesion molecule-A [JAM-1], claudin-1, claudin-2, and claudin-4 contain 4%, 8%, 4%, and 3% Tyr, respectively) (28, 29), providing the possibility that occludin could be a readily available surface target for MPO.

Based on this analysis, we sought to define the extent of Tyr residue chlorination on epithelial occludin in the presence of PMN activation. To do this, we immunoprecipitated occludin and performed EC-HPLC analysis of Tyr and 3-Cl-Tyr following pronase digestion (Figure 5B). We analyzed occludin isolated from T84 cells exposed to inactivated PMNs and activated PMNs (Figure 5, C–E). As shown in Figure 5D, we observed a distinct 3-Cl-Tyr peak (retention time ~4.8 minutes) in samples exposed to activated PMNs but not in those exposed to inactivated PMNs (Figure 5C). As shown in Figure 5E, chlorinated Tyr was increased by more than 6-fold (*P* < 0.05) in T84 intestinal epithelial cells (IECs) exposed to activated PMNs compared with inactivated PMNs. No significant 3-Cl-Tyr was detected in T84 cells alone. To determine the role of MPO in the chlorination of occludin Tyr residues, we exposed T84 and Caco-2 IECs to recombinant MPO or activated recombinant MPO (MPO in the presence of low pH and hydrogen peroxide) and examined occludin Tyr chlorination. In both T84 and Caco-2 IECs, 3-Cl-Tyr was detected within occludin at levels greater than 3- to 4-fold over controls (Figure 5F).

To determine if the observed Tyr chlorination was specific for occludin, we analyzed other TJ proteins containing extracellular domains. We immunoprecipitated both claudin-1 and JAM-1 from colonic tissue of WT and MPO-KO mice that had undergone chronic DSS. As shown in Figure 5G, in both claudin-1 and JAM-1, 3-Cl-Tyr was detected in samples derived from WT but not MPO-KO mice treated with DSS (*P* < 0.01). It is notable that the magnitude of Tyr chlorination was less in claudin-1 and JAM-1 than occludin, tracking with fewer Tyr residues in the extracellular domains (28, 29). There was no significant Tyr chlorination observed in WT and MPO-KO mice treated with water (Supplemental Figure 5D). In the WT mice, when comparing DSS-treated mice and water controls, there was increased Tyr chlorination in occludin, claudin-1, and JAM-1. This increase in 3-Cl-Tyr was not observed in the MPO-KO mice.

As an additional level of analysis, we extended these studies to examine the specificity of Tyr chlorination to transmembrane proteins. To do this, we immunoprecipitated zonula occluden-1 (ZO-1), an intracellular TJ protein, and analyzed the protein for 3-Cl-Tyr (30, 31). T84 IECs were exposed to both activated PMNs and activated MPO before immunoprecipitating and digesting ZO-1 for analysis of 3-Cl-Tyr. As shown in Figure 5G, neither activated PMNs or activated MPO chlorinated Tyr within ZO-1, as was observed in occludin (Figure 5, E and F). These results suggest that chlorination of Tyr by MPO is specific to proteins that contain extracellular domains.

### Chlorination of the occludin increases epithelial permeability.

**Figure 6:**
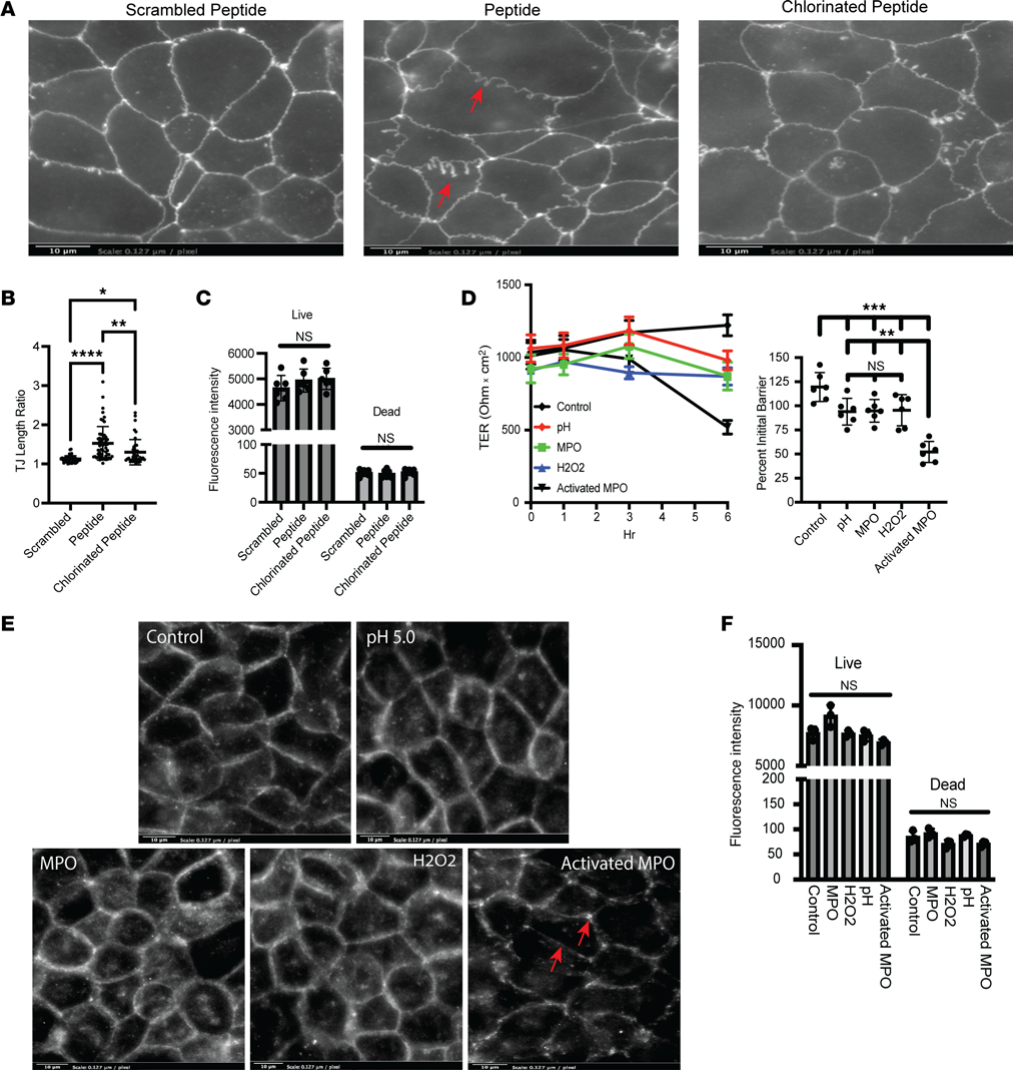
Activated MPO disrupts the epithelial barrier. (**A**) Representative immunofluorescence images of ZO-1 in Caco-2 IECs treated with 200 μg/mL scrambled, nonchlorinated, or chlorinated occludin peptide for 6 hours. Arrows mark regions of mislocalization or decreased expression. (**B**) Analysis of the TJ ratio in Caco-2 IECs treated with 200 μg/mL scrambled, nonchlorinated, or chlorinated occludin peptide for 6 hours. More than 30 total TJs were measured across biological replicates. A total of 3 biological replicates (cells cultured at different time points) used. (**C**) Caco-2 IECs were incubated with 200 μg/mL of scrambled, nonchlorinated, or chlorinated occludin peptide for 24 hours. After 24 hours the cells were analyzed for cell death using a fluorescence-based live/dead assay. (**D**) Transepithelial electrical resistance (TER) values over time and percentage initial TER in Caco-2 IECs following 6 hours’ exposure to control (pH 7.4), pH 5.0, 1 μg/mL MPO, 200 μM H_2_O_2_, or activated MPO, a combination of pH 5.0/MPO/H_2_O_2_, for 6 hours on both the apical and basolateral surfaces. Data represent 6 biological replicates, each replicate. (**E**) Representative immunofluorescence images of occludin in Caco-2 IECs following 6 hours’ exposure to control, pH 5.0, 1 μg/mL MPO, 200 μM H_2_O_2_, or activated MPO. Arrows indicate regions of aberrant occludin staining, loss of signal, and formation of distinct puncta. (**F**) Caco-2 IECs were incubated for 6 hours in the presence of low pH, H_2_O_2_, MPO, or a combo. After 6 hours the cells were analyzed for cell death using a fluorescence-based live/dead assay. Data are expressed as mean ± SD, and the *P* value was determined by 1-way ANOVA. *P < 0.05, **P < 0.01, ***P < 0.001, ****P < 0.0001.

The second extracellular loop of occludin is required for fully functional TJ complex in multiple species (32, 33). In human endothelial cells, the function of occludin has been studied using specific cyclic 6-mer peptides derived from the second extracellular loop domain of occludin (34). This 6-mer peptide sequence contains 2 Tyr residues. We sought to examine the impact of chlorination of these Tyr residues on occludin-occludin binding. Caco-2 IECs were incubated with a scrambled peptide, 6-mer peptide, or chlorinated 6-mer peptide for 6 hours followed by analysis of TJ morphology. As shown in Figure 6A, Caco-2 IECs exposed to the occludin peptide revealed the appearance of an abnormal, undulating morphology and punctate staining of the TJ marker ZO-1 (marked by arrows), indicating the 6-mer peptide was disrupting the native binding of cellular occludin. We quantified the junctional length relative to a straight line between tricellular junctions (length ratio, analysis depicted in Figure 6B). This TJ length ratio has been shown to be a reasonable indicator of TJ integrity (35). This analysis revealed a significant increase in TJ length ratio in Caco-2 IECs treated with the occludin peptide when compared with a scrambled peptide (*P* < 0.0001). When the Tyr residues were chlorinated on the peptide, the TJ length ratio was decreased when compared with the nonchlorinated occludin peptide, indicating that chlorination of the Tyrs in the 6-mer peptides prevented the interaction of the peptide with cellular occludin (*P* < 0.0025). We next examined if the change in TJ morphology and length was a result of increased cell death. As shown in Figure 6C, 24-hour treatment with the scrambled, nonchlorinated, or chlorinated peptide had no impact on cell viability. This observation suggests that chlorination of the Tyr within the occludin/occludin binding site prevented the 6-mer peptide from disrupting the native occludin-occludin binding. These observations were verified in T84 IECs, in which treatment with the occludin peptide resulted in longer ZO-1 TJ ratios when compared with the scrambled peptide and chlorinated occludin peptide (Supplemental Figure 6B, *P* < 0.0001). We extended our analysis of the TJ to include TJ ratio of occludin, in a manner similar to the ZO-1 TJ ratio above. In T84 IECs the occludin peptide increased the TJ ratio of occludin when compared with TJ ratios of T84 IECs treated with the chlorinated occludin peptide (*P* < 0.001). Therefore, these studies indicate that chlorinated Tyr residues within the occludin peptide structure significantly disrupt intact TJ structure.

### Activated MPO disrupts epithelial barrier function.

Activated MPO promotes bystander tissue damage and inhibits wound healing (18). We examined TJ barrier function following exposure to activated recombinant MPO at neutral and acidic pH in the presence of H_2_O_2_. As shown in Figure 6D, there was a significant decrease in Caco-2 IEC transepithelial electrical resistance (TER) following a 6-hour exposure to activated MPO when compared with pH 7.4 controls or Caco-2 treated with pH 5.0, H_2_O_2_, or MPO only (*P* < 0.001). After 6 hours the inserts were collected, fixed, and stained for occludin. As shown in Figure 6E, there was a decrease in occludin staining in the Caco-2 IECs treated with activated MPO when compared with all other conditions. Similar observations were made in the T84 IEC line with occludin and in the Caco-2 IECs stained for ZO-1 (Supplemental Figure 7, A and C). Thus, changes in IEC TJ structure and function can be recapitulated with recombinant MPO. The impact of low pH, H_2_O_2_, and activated MPO on IEC viability was determined by a live/dead cell assay (Figure 6F). None of the treatment conditions negatively influenced cell viability in the Caco-2 IECs. Similar results were observed in T84 IECs exposed to the same conditions (Supplemental Figure 7B).

### Increased 3-Cl-Tyr on occludin in patients with active UC.

**Figure 7:**
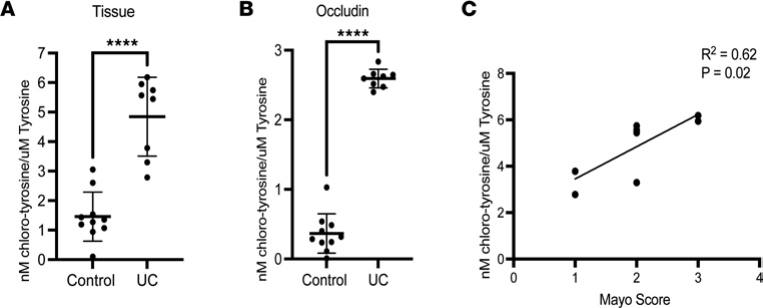
Tyrosine chlorination in human patients with UC. (**A**) Analysis of Tyr chlorination from whole tissue isolated from healthy controls and patients with UC. (**B**) Analysis of Tyr chlorination within occludin isolated from healthy and UC patients. (**C**) Correlation between tissue chlorinated Tyr and Mayo scores in individuals with UC. Data are expressed as mean ± SD, and the *P* value was determined by *t* test. ****P < 0.0001.

Finally, we extended these studies to include tissue samples from human patients with active colitis. A comparison of healthy controls (*n* = 10) and active UC patient biopsies (*n* = 8) with variable severity of inflammation (Table 1) revealed an overall increase in 3-Cl-Tyr in individuals with active UC when compared with uninflamed controls (Figure 7A, *P* < 0.0001). In further analysis, we examined whether occludin Tyr was chlorinated in biopsies from patients with actively inflamed UC. As shown in Figure 7B, and consistent with our in vitro and mouse colitis model, occludin 3-Cl-Tyr from individuals with active UC was significantly increased compared with healthy controls (*P* < 0.0001). Moreover, the degree of occludin Tyr chlorination correlated with the severity of macroscopic disease as determined by the Mayo score (*R*^2^ = 0.62, *P* < 0.025) (Figure 7C). These findings implicate occluding Tyr chlorination by MPO as a potential mechanistic link to barrier dysfunction and attenuated inflammatory resolution in the mucosa of patients with UC.

## Discussion

Accumulation of PMNs at sites of inflammation, as observed in inflammatory bowel disease (IBD), represents a prototypical double-edged sword, where PMNs are essential for innate immunity but are also associated with bystander tissue damage (36). While PMN-mediated bystander tissue damage likely occurs through more than one mechanism, oxidative processes and the release of proteolytic enzymes appear to be primary culprits (1, 36). Despite a long history of research, surprisingly little is known about the mechanisms of MPO-mediated host tissue damage. Given the significant presence of MPO at sites of inflammation and its role in bystander tissue damage, we sought to examine the molecular mechanisms of MPO action on the development of chronic inflammation and associated MPO-mediated mucosal damage.

As the most abundant enzyme in the PMN, MPO catalyzes the formation of HOCl from H_2_O_2_ and Cl ion. HOCl is a potent and highly reactive oxidant (3, 4, 37). MPO and HOCl have been shown to promote pathology in several diseases, including IBD, where MPO inhibits wound healing and promotes inflammation in the colonic mucosa (16, 18, 38). Original studies in MPO-deficient human patients revealed candidiasis as the primary disease presentation and revealed that MPO-deficient PMN function was relatively normal, with some slowed kinetics in killing *Staphylococcus* *aureus* (39). Subsequent studies revealed that MPO-deficient PMNs show extended respiratory burst rates and that the peroxidase activity of MPO may be more important in the termination of the respiratory burst (40). We found that the loss of MPO significantly enhanced the resolution of DSS-induced colitis. MPO-KO mice had less inflammation when compared with WT mice, consistent with a previous study (16), and the inflammation that was present resolved more rapidly. Fecal lipocalin served as an excellent biomarker after DSS was removed and remained high in WT but not MPO-KO mice. It is noteworthy that DSS colitic mice express sepsis-like symptoms (41) and that mice deficient in enzymes necessary for the respiratory burst (e.g., gp91) show significantly increased susceptibility to DSS colitis (20), suggesting that MPO may be redundant as an antimicrobial in mucosal inflammation. It is for this reason that we focused on the role of MPO in bystander tissue damage and chronic inflammation.

Using an acute and chronic model of DSS colitis, we observed a progressive decrease in disease severity in mice lacking MPO. During PMN migration and activation, MPO is released into the extracellular space where it primarily catalyzes H_2_O_2_ to HOCl. It is noteworthy that MPO can act as a signaling molecule independent of its enzymatic activity (3, 4, 10, 42, 43). Given that MPO has nonenzymatic functions, we sought to determine the impact of enzymatic inhibition of MPO on acute and chronic colitis. We used the microbiota-derived MPO inhibitor, IPA, to examine differences in MPO inhibition as a comparator to genetic deletion of MPO on the development of acute and chronic colitis. In WT mice, IPA administration decreased disease severity in both acute and chronic models of colitis. In the MPO-KO mice, administration of IPA had no impact on disease severity in both the acute and chronic model.

A unique feature of MPO is the production of HOCl, a potent 2-electron oxidant that diffuses through the tissue and reacts with sulfur and nitrogen atoms. Within the tissue, HOCl reacts indiscriminately with Tyr to create 3-Cl-Tyr, a heat-stable and reliable marker of HOCl oxidation and in vivo marker of MPO activity (3, 5–7). As proof of principle, MPO-KO mice showed little to no detectable 3-Cl-Tyr following DSS treatment. An interesting observation is that the 2 extracellular loops of occludin, essential for TJ function, contain a disproportionately high number of Tyr residues compared with other transmembrane TJ proteins and serve as potential targets for MPO. Our studies show that both activated PMNs and activated recombinant MPO chlorinate Tyr within occludin. These in vitro observations were supported by patient samples. 3-Cl-Tyr was observed in occludin from patients with active UC but not observed in healthy controls. Furthermore, analysis of intracellular TJ protein, ZO-1, revealed no significant increase in 3-Cl-Tyr following exposure to activated PMNs or activated recombinant MPO when compared to controls, suggesting that chlorination of amino acids is specific to the extracellular space. This observation was supported by the observation that both claudin-1 and JAM-1, 2 other TJ proteins with extracellular domains, also showed significant Tyr chlorination in active colitis (Figure 5G).

It has been reported that the second extracellular loop of occludin is relatively more important for occludin-to-occludin interaction and TJ function than the first extracellular loop (32, 33). Interestingly, upon closer examination of the second extracellular loop, the peptide sequence associated with occludin-occludin interaction contains 2 Tyr residues in close proximity (34). Three lines of evidence provide insight into this mechanism. First, in cultured epithelia, activated MPO prominently induced a loss of barrier function. Second, analysis of immunoprecipitated occludin revealed prominent chlorination of Tyr in epithelial cells exposed to activated PMNs. Third, disrupting peptides corresponding to the active interface of occludin-occludin interactions lost their disruptive capacity upon the chlorination of Tyr within the peptides. Collectively, these results indicate that Tyrs in the extracellular loops of occludin become targets for chlorination by MPO to disrupt epithelial barrier function. The disruption of occludin-occludin interaction via chlorination of Tyr may contribute to the decrease in occludin observed in patients with UC along with cytokine signaling and bystander tissue damage.

In summary, our findings reveal that PMN MPO significantly contributes to the development of chronic inflammation and is another important aspect of the inflammatory microenvironment. Furthermore, we demonstrate that the inhibition of MPO utilizing a bacterially derived MPO inhibitor act in a similar manner as genetic deletion of MPO. These results suggest that it is the enzymatic action of MPO that promotes inflammation and not the presence of MPO in the tissue. This observation is supported by the prior observations that the enzymatic activity of MPO has been reported to be damaging to the tissue and to inhibit wound healing in both in vitro and in vivo models (18). Based on the observation that chlorination within the occludin binding domain disrupts TJ function and that Tyr chlorination occurs indiscriminately at sites of inflammation, more research into the impact of Tyr chlorination on cellular function is needed (44). Adding to the complexity of MPO impact on the inflammatory microenvironment, it has been reported that MPO has various nonenzymatic functions during inflammation (45). MPO can be taken up by macrophages through the mannose receptor, which increases their activation and cytokine secretion (46). It has also been shown that MPO can activate endothelial cells to produce Il-6 and Il-8 (47). Taken together these studies highlight the need to further study the impact of MPO on the inflammatory microenvironment to better understand all mechanisms by which MPO promotes inflammation and alterations to the TJ.

## Methods

### Sex as a biological variable.

Both male and female mice were used at equal numbers in all animal studies. Sex was not considered as a biological variable, and differences between male and female mice were not analyzed.

### Materials availability.

This study did not generate new unique reagents.

### Cell culture.

T84 (CCL-248; American Type Culture Collection, ATCC) and Caco-2 C2BBe1 (CRL-2102; ATCC) human epithelial cell lines were obtained and cultured in 95% air with 5% CO_2_ at 37°C according to instructions provided by ATCC. Low-passage (<20) cells were cultured on 0.4 μm Transwell inserts (Corning) for 7–10 days to obtain confluent cell monolayers, determined by TER, greater than 600 Ohm × cm^2^, using a commercially available voltage clamp (EVOHM2, World Precision Instruments). Cell viability was determined using the Live/Dead Cell Viability Assay Kit (catalog ab287858, Abcam) following manufacturer’s instructions. At confluence T84 and Caco-2 IECs were treated with control (pH 7.4 HBSS), pH 5.0 HBSS, 1 μg/mL MPO (R&D Systems), 200 μM H_2_O_2_ (Thermo Fisher Scientific), or activated MPO (combination of pH 5.0/MPO/H_2_O_2_). The cells were collected for immunofluorescence, TER analysis, or chlorinated Tyr analysis, as described below.

### Animal studies.

WT C57BL/6 and MPO-KO mice (The Jackson Laboratory) were bred in-house. Sex-, age-, and weight-matched mice were used in DSS studies. Mice were 8–12 weeks of age at the start of the experiments. Induction of colitis in mice with colitogenic DSS was performed as previously described (5, 17, 19, 48). DSS (~40,000 MW; Chem-Impex) was added to drinking water (2.5%) for 5 days before being replaced with water, followed by monitoring for weight loss over 7 days. For chronic DSS, 2.5% DSS was added to drinking water for 5 days before being replaced with water, followed by monitoring for weight loss over 7 days. The mice were allowed to recover for 16 days before adding 2.5% DSS to the drinking water for another 5 days. This was repeated for a third round. Tissue was collected in the chronic DSS model 2 days after DSS was removed. For IPA studies, mice were given 0.1 mg/mL IPA in drinking water throughout the duration of the experiment, including between rounds of DSS. Cohorts of 4–6 mice were collected after the first, second, and third rounds of DSS. Postmortem colons were harvested by blunt dissection. The colons were separated longitudinally and half of the colon was collected and fixed in 10% buffered formalin (MilliporeSigma) prior to paraffin-embedding and staining with H&E. From the other half of colon tissue, in some mice the distal 2 cm was collected for either RNA and protein isolation or for analysis of Tyr and chlorinated Tyr. In the other mice the half of the colon was collected for analysis of neutrophil infiltration by flow cytometry.

### Patient samples.

Endoscopic disease status and location were noted by the physician who obtained the biopsy samples collected from the University of Colorado Anschutz Medical Campus. A total of 10 healthy controls and 8 inflamed UC samples (collected from both the colon and rectum) were analyzed for 3-Cl-Tyr by EC-HPLC.

### PMN isolation and stimulation.

Human neutrophils were isolated from whole venous blood of healthy volunteers as described in detail previously (49) (IRB 06-0853). Briefly, whole venous blood was collected in syringes containing anticoagulant (K_2_EDTA at 1.8 mg/mL blood). Blood was gently layered over double-density Histopaque gradients (1119/1077) and centrifuged at 700*g* in a swinging bucket rotor centrifuge for 30 minutes without brake. The resulting granulocyte layer was collected, and residual red blood cells were lysed. PMNs were washed with ice-cold HBSS (with/without CaCl_2_ or MgCl_2_), counted, and used within 2 hours of isolation.

### Histological scoring.

Colon samples were fixed in 10% neutral buffered formalin and paraffin-embedded before staining for H&E. All histologic quantitation was performed masked, by the same individual, using a scoring system as previously described (50). Briefly, the 3 independent parameters measured were severity of inflammation (0 to 3: none, slight, moderate, severe), extent of injury (0 to 3: none, mucosal, mucosal and submucosal, transmural), and crypt damage (0 to 4: none, basal 1/3 damaged, basal 2/3 damaged, only surface epithelium intact, entire crypt and epithelium lost). The score of each parameter was multiplied by a factor reflecting the percentage of tissue involvement (×1: 0% to 25%, ×2: 26% to 50%, ×3: 51% to 75%, ×4: 76% to 100%), and all numbers were summed. The maximum possible score was 40.

### Immunofluorescence.

To localize ZO-1 and occludin expression in IECs, Caco-2 and T84 IECs were plated on 0.33 cm^2^ permeable supports (0.4 μm pore, Corning) until confluent, as measured by TER. Inserts were washed with HBSS containing CaCl_2_ and MgCl_2_ (HBSS+) and fixed with iced 1:1 acetic acid/methanol for 15 minutes at 4°C. After fixation, inserts were briefly washed with phosphate-buffered saline (PBS, Thermo Fisher Scientific). Inserts were blocked in PBS containing 10% goat serum (Jackson ImmunoResearch) at room temperature (RT) for 1 hour. Inserts were stained with rabbit polyclonal anti–ZO-1 (catalog 40-2200, Invitrogen) or rabbit monoclonal anti-occludin (catalog ab216327, Abcam) followed by Alexa Fluor 568 secondary Ab (catalog A11011, Invitrogen) and counterstained with ProLong Gold Antifade with DAPI (Thermo Fisher Scientific). Inserts were imaged using an EVOS M5000 equipped with DAPI and RFP light cubes and an EVOS ×40 fluorite objective (Thermo Fisher Scientific).

### Occludin peptide.

We examined the impact of Tyr chlorination on occludin binding using a synthetic peptide targeting the occludin binding site (cyclic H2N-CLYHYC-OH), a chlorinated occludin binding peptide [cyclic H2N-CL(3ChloroY)H(3ChloroY)C-OH], and a scramble cyclic peptide (cyclic H2N-YCHLYC-OH) at 500 μg/mL for 24 hours (Biosynth International).

### Analysis of fecal lipocalin.

To examine fecal lipocalin 2, fecal pellets from WT and MPO-KO mice were collected before the start of DSS and throughout the development and resolution of colitis. The fecal pellets were weighed and reconstituted in PBS containing 0.1% Tween 20 (50 mg/mL) and vortexed for 20 minutes to get a homogenous fecal suspension. The samples were then centrifuged for 10 minutes at 12,000 rpm and 4°C. Clear supernatants were collected and stored at –20°C until analysis. Lipocalin 2 levels were estimated in the supernatants using murine lipocalin-2 ELISA kit (RayBiotech).

### RNA isolation and qPCR.

Total RNA was extracted from cells using TRIzol (Invitrogen) and from tissue using RNeasy Mini Kit (QIAGEN). cDNA was prepared using iScript cDNA synthesis kit (Bio-Rad). qPCR to measure transcripts was performed in 1× Power SYBR Green Master Mix (Applied Biosystems) using an ABI 7300 thermocycler (Applied Biosystems). Fold-change in expression of target mRNA relative to β-actin mRNA was calculated as previously described (51). Primer sequences are listed in Table 2.

### Analysis of inflammatory markers using MESO scale.

Distal colon samples (50 mg) were homogenized for 10 seconds in lysis buffer (150 mM NaCl, 20 mM Tris [pH 7.5], 1 mM EDTA, 1 mM EGTA, 0.1% SDS, protease inhibitor mixture) (Roche) and phosphatase inhibitors (MilliporeSigma) at 4°C. Samples were cleared by centrifugation, and the protein concentration was determined by BCA assay (Pierce, Thermo Fisher Scientific). Supernatants were assayed for indicated cytokines by the chemiluminescence-based sandwich immunoassay, V-PLEX Mouse Cytokine 19-Plex kit (catalog K15255G, Meso Scale Diagnostics), according to the manufacturer’s protocols. Briefly, the assay plates were washed before adding 50 μL of sample and incubating at RT for 3 hours. The plate was washed 3 times, the detection antibodies were added, and the plate was incubated for 2 hours at RT. The plate was then washed 3 times before being analyzed on a Sector 2400 Imager (Meso Scale Diagnostics). Chemokine and cytokine concentrations were normalized using the calculated protein concentrations.

### Analysis of tyrosine chlorination activity by EC-HPLC.

Tyr and 3-Cl-Tyr were quantified using isocratic reversed-phase HPLC with electrochemical coulometric array detection (EC-HPLC) (CoulArray, Thermo Fisher Scientific). Separation was achieved using an Acclaim Polar Advantage II C18 column, 5 μm 120 Å, 4.6 × 150 mm (Thermo Fisher Scientific), at a flow rate of 0.6 mL/min in a mobile phase consisting of 10% acetonitrile in 50 mM sodium phosphate buffer, pH 3, containing 0.42 mM octanesulphonic acid as an ion-pairing agent. The data were collected and quantified using the CoulArray software with comparison to Tyr (MilliporeSigma) and 3-Cl-Tyr (Alfa Aesar) standards. For the analysis of MPO chlorination in vivo, colon tissue from DSS-treated WT and MPO-KO mice and mice treated with DSS and either with/without IPA were sonicated and digested overnight at 37°C using 5 mg/mL of Pronase (MilliporeSigma 10165921001). The digests were then spun to pellet insoluble material, and the resulting supernatants were filtered through spin columns (5 kDa molecular weight cutoff, Sartorius VS0112) prior to HPLC analysis.

### Immunoprecipitation.

Chlorination of Tyr within occludin, ZO-1, claudin-1, and JAM-1 was determined by EC-HPLC analysis on immunoprecipitated proteins from murine tissue and in vitro T84 and Caco-2 IECs. Tissue and cells were collected in RIPA lysis buffer containing protease and phosphatase inhibitors and briefly sonicated, 20–30 seconds, until the tissue was fully disrupted. The lysates were spun to pellet insoluble material and the supernatants collected for immunoprecipitation. The lysates were precleared by adding 50 μL goat serum and incubating for 1 hour at 4°C. A total of 100 μL of Pierce Protein A/G agarose (Thermo Fisher Scientific) was added to each sample and then incubated for 30 minutes at 4°C with gentle agitation. Following incubation the solution was centrifuged at 14,000*g* for 10 minutes and the supernatant collected. Between 50 and 100 μg of cell lysate was incubated with 2 μg of ZO-1 (catalog 40-2200, Invitrogen), Claudin-1 (catalog ab211737, Abcam), JAM-1 (catalog ab269948, Abcam), and occludin (catalog ab216327, Abcam) antibody for 16 hours at 4°C with gentle rotation. After incubation 200 μL of Pierce Protein A/G agarose (Thermo Fisher Scientific) was added to each sample and incubated for 4 hours at 4°C under gentle rotation. Samples were washed 3 times with RIPA before the agarose beads were digested overnight at 37°C using 5 mg/mL of Pronase (MilliporeSigma 10165921001). The digests were then spun to pellet insoluble material, and the resulting supernatants were filtered through spin columns (5 kDa molecular weight cutoff) prior to HPLC analysis as described above.

### Statistics.

Statistical analysis was performed using GraphPad Prism version 9.5.1 (733) Data were assessed for normal distribution using the Shapiro-Wilk test. For data with normal distribution, the appropriate parametric test was used, and an appropriate nonparametric test was used when data was not normally distributed. Depending on the data, an unpaired 2-tailed *t* test (with Welch’s correction where variance was unequal), Mann-Whitney *U* test, ordinary 1-way ANOVA (Welch’s ANOVA where variance was unequal using a Brown-Forsythe test), or Kruskal-Wallis test was used. For data analyzed with an ordinary 1-way ANOVA, Tukey’s multiple comparisons test was used to compare groups. When Kruskal-Wallis test was used, Dunn’s multiple comparisons test was used to perform multiple comparisons between groups. Results were deemed statistically significant when *P* < 0.05.

### Study approval.

All animals were handled according to protocols approved by the institutional committee for animal use (CU Anschutz IACUC Protocol 00182). Healthy and UC patient biopsies were collected from the University of Colorado Crohn’s and Colitis Center with institutional review board approval (IRB 14-2012). Patient written consent was obtained prior to the procedure. During the consent process patients were informed on how the tissue would be used after collection.

### Data availability.

Underlying data are available from the corresponding author upon request and in the Supporting Data Values XLS file.

## Author contributions

IMC and SPC designed experiments, provided funding, analyzed data, wrote the manuscript, and provided supervision. LZ, SDK, NW, DZ, and RC performed experiments and analyzed data. CAS, MEG, and JCO provided materials and reagents.

## Supplementary Material

Supplemental data

Supporting data values

## Figures and Tables

**Table 1:**
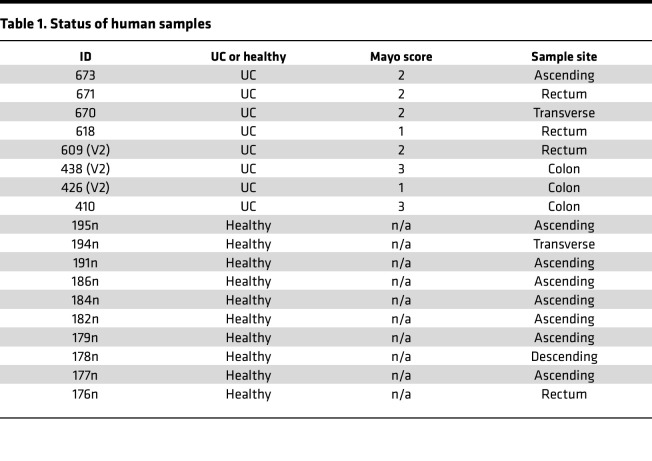
Status of human samples

**Table 2:**
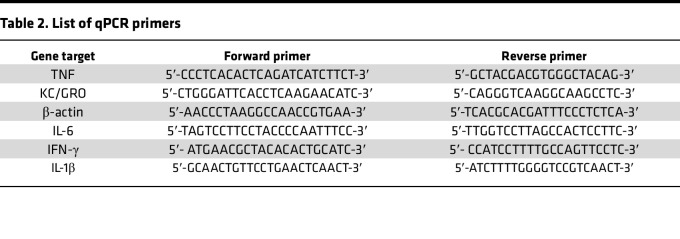
List of qPCR primers

## Version 1 07/22/2024

Electronic publication
