# Supplementary material for: Chlorination of epithelial tight junction proteins by neutrophil myeloperoxidase promotes barrier dysfunction and mucosal inflammation
Source: JCI Insight. 2024 Jun 11;9(14):e178525. doi: 10.1172/jci.insight.178525 (PMC11383587; doi:10.1172/jci.insight.178525)
Supplement: Supplemental data [file jciinsight-9-178525-s100.pdf]

Supplemental 1: qPCR analysis of pro-inflammatory genes in acute DSS colitis. (A) MESO scale analysis of proinflammatory cytokines in the distal colon of WT and MPO KO mice on day 7, 2 days after DSS was removed. (B) qPCR analysis of proinflammatory cytokines in the distal colon of WT and MPO KO mice following acute DSS colitis. (C) MESO scale analysis of proinflammatory cytokines in the distal colon of WT and MPO KO mice at Day 21, 16 days after DSS was removed. n = 3-4 mice per group panel A and C. n = 8-10mice per group b. Data is expressed as mean $\pm$ SD and the *p*-value determined by T-test (B, C) or 1-Way ANOVA (A). $*p < 0.05$, $** p < 0.01$, $*** p < 0.001$.

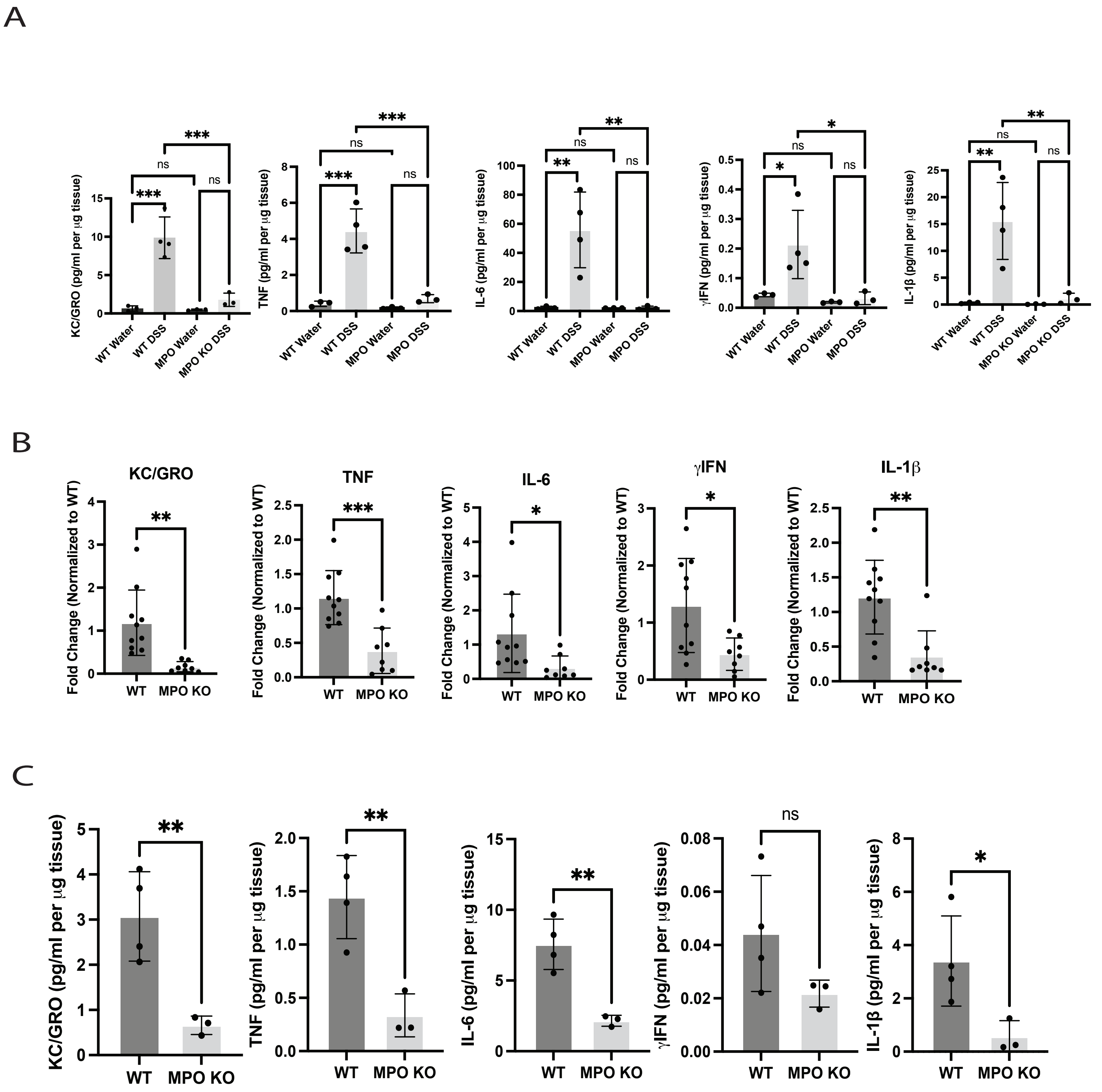
A
KC/GRO (pg/ml per μg tissue)
TNF (pg/ml per μg tissue)
IL-6 (pg/ml per μg tissue)
γIFN (pg/ml per μg tissue)
IL-1β (pg/ml per μg tissue)
WT Water
WT DSS
MPO Water
MPO KO DSS
MPO DSS
MPO KO Water
ns
*
**
***
B
KC/GRO
TNF
IL-6
γIFN
IL-1β
Fold Change (Normalized to WT)
WT
MPO KO
C
KC/GRO (pg/ml per μg tissue)
TNF (pg/ml per μg tissue)
IL-6 (pg/ml per μg tissue)
γIFN (pg/ml per μg tissue)
IL-1β (pg/ml per μg tissue)
WT
MPO KO

Supplemental 2: qPCR analysis of pro-inflammatory genes in chronic DSS colitis. (A) Fecal blood scores from WT and MPO KO mice during each round of DSS colitis. (B and C) qPCR analysis of proinflammatory cytokines in the distal colon of WT and MPO KO mice following the second and third round of DSS colitis. n= 6-14 mice per group panel A, n = 4-5 mice per group panel B, and n = 11 mice per group panel C. Data is expressed as mean ± SD and the *p*-value determined by T-test (B, C) or 2-Way ANOVA (A) were appropriate. $^*p < 0.05$, $^{**} p < 0.01$.

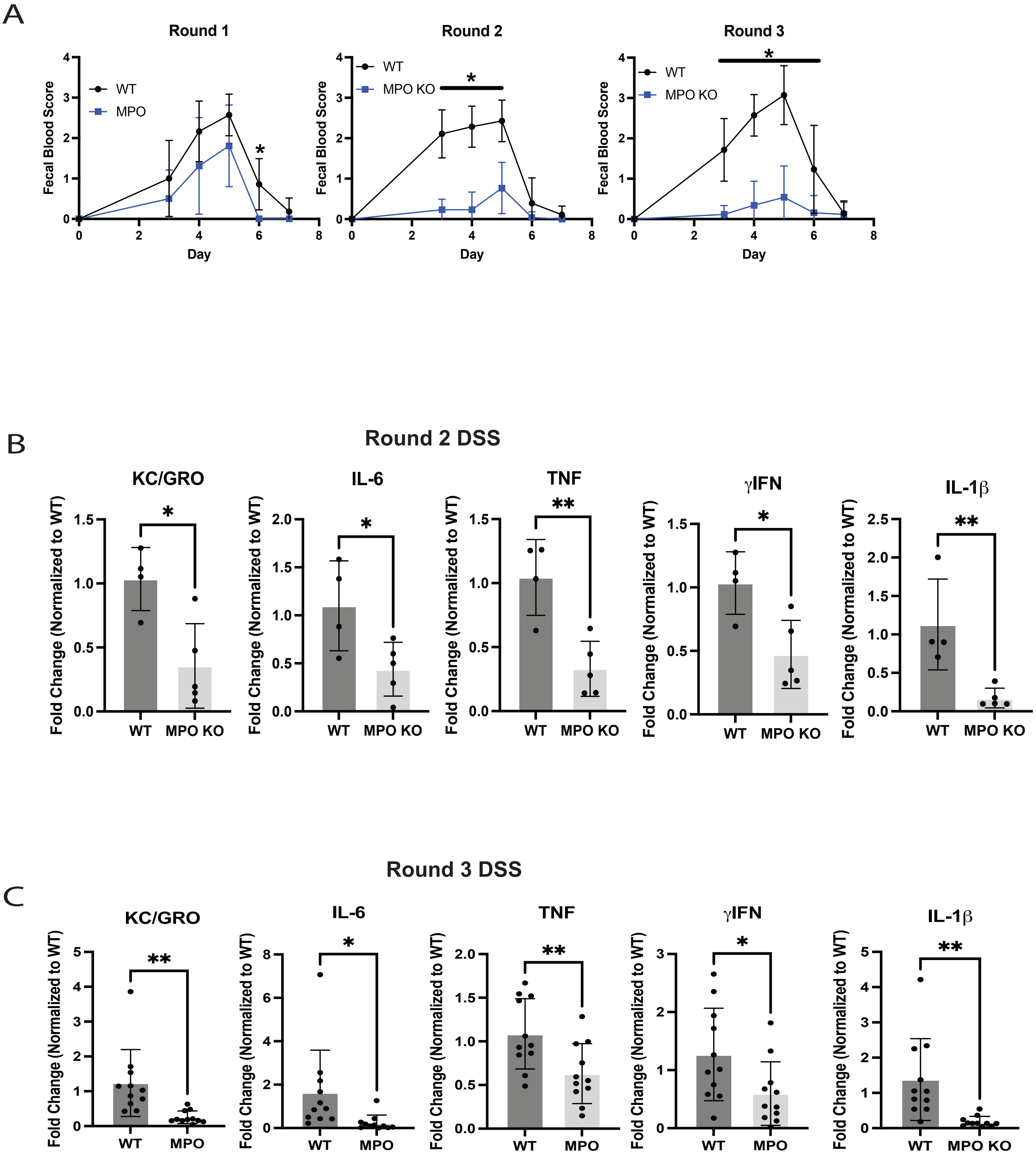
A
Round 1
Round 2
Round 3
Fecal Blood Score
WT
MPO
MPO KO
*
Day
B
Round 2 DSS
KC/GRO
IL-6
TNF
γIFN
IL-1β
Fold Change (Normalized to WT)
WT
MPO KO
*
**
C
Round 3 DSS
KC/GRO
IL-6
TNF
γIFN
IL-1β
Fold Change (Normalized to WT)
WT
MPO
MPO KO
*
**

Supplemental 3: Analysis of PMN infiltrate in acute and chronic DSS colitis. Flow cytometry analysis of tissue PMN in water control (A) and after the 1st, 2nd, and 3rd round of DSS colitis (B) in WT and MPO KO mice. n = 5-10 mice per group. (C) Analysis of granulomas, normalized to colon length, in MPO KO and WT mice treated with 1 (acute) or 3 (chronic) rounds of DSS. n = 10-24 mice. Data is expressed as mean $\pm$ SD and the *p*-value determined by T-test (B) or 1-Way ANOVA (A, C). * $p < 0.05$.

A

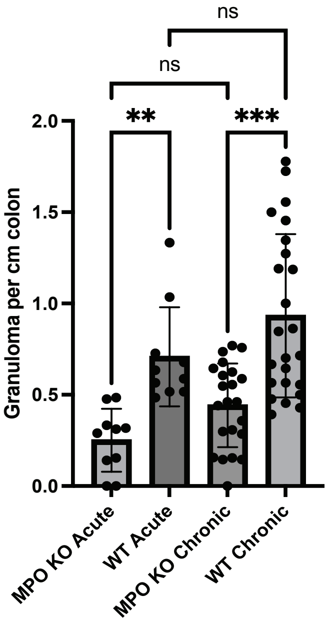
ns
ns
**
***
Granuloma per cm colon
2.0
1.5
1.0
0.5
0.0
MPO KO Acute
WT Acute
MPO KO Chronic
WT Chronic

B

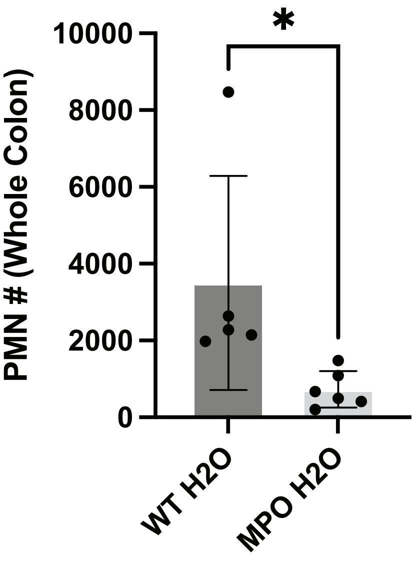
*
PMN # (Whole Colon)
10000
8000
6000
4000
2000
0
WT H2O
MPO H2O

C

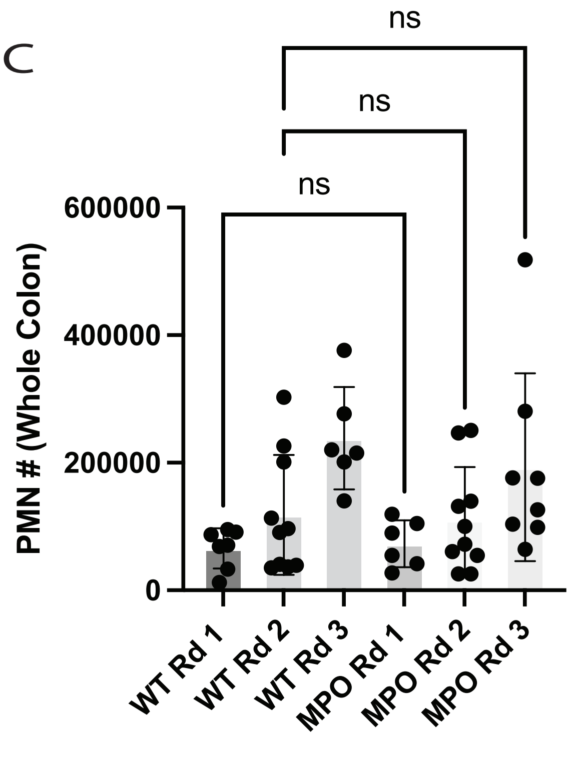
ns
ns
ns
PMN # (Whole Colon)
600000
400000
200000
0
WT Rd 1
WT Rd 2
WT Rd 3
MPO Rd 1
MPO Rd 2
MPO Rd 3

Supplemental 4: qPCR analysis of pro-inflammatory genes in IPA treated mice. qPCR analysis of proinflammatory cytokines in the distal colon of WT and MPO KO mice treated with 0.1 mg/mL IPA in drinking water following chronic DSS colitis. n = 3-12 mice per group. Data is expressed as mean $\pm$ SD and the *p*-value determined by 1-Way ANOVA. $^*p < 0.05$, $^{**}p < 0.01$, $^{***}p < 0.001$, $^{****}p < 0.0001$.

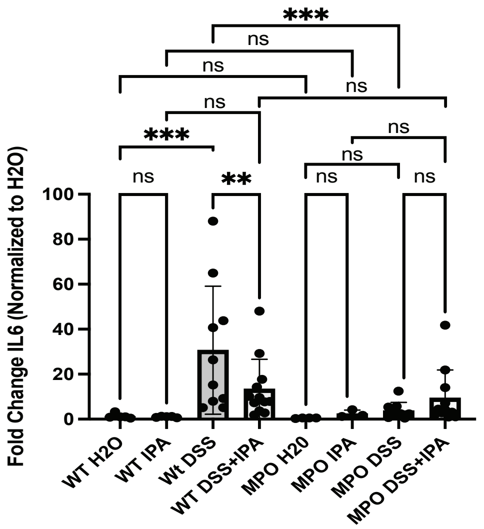

Fold Change IL6 (Normalized to H2O)
0
20
40
60
80
100
WT H2O
WT IPA
Wt DSS
WT DSS+IPA
MPO H20
MPO IPA
MPO DSS
MPO DSS+IPA
***
ns
ns
ns
ns
***
ns
**
ns
ns
ns
ns
ns

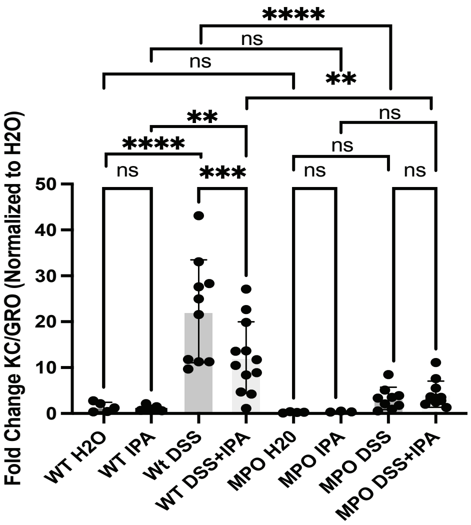

Fold Change KC/GRO (Normalized to H2O)
0
10
20
30
40
50
WT H2O
WT IPA
Wt DSS
WT DSS+IPA
MPO H20
MPO IPA
MPO DSS
MPO DSS+IPA
****
ns
ns
**
**
ns
****
ns
ns
***
ns
ns

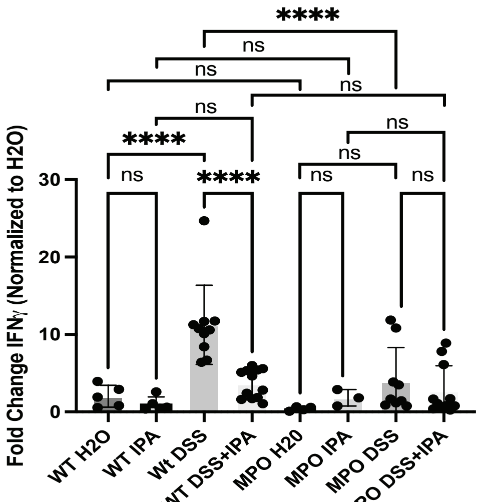

Fold Change IFNγ (Normalized to H2O)
0
10
20
30
WT H2O
WT IPA
Wt DSS
WT DSS+IPA
MPO H20
MPO IPA
MPO DSS
MPO DSS+IPA
****
ns
ns
ns
ns
****
ns
****
ns
ns
ns
ns

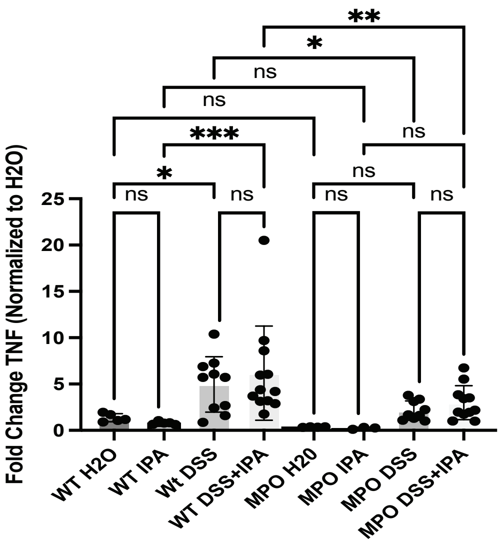

Fold Change TNF (Normalized to H2O)
0
5
10
15
20
25
WT H2O
WT IPA
Wt DSS
WT DSS+IPA
MPO H20
MPO IPA
MPO DSS
MPO DSS+IPA
**
*
ns
ns
***
ns
*
ns
ns
ns
ns
ns

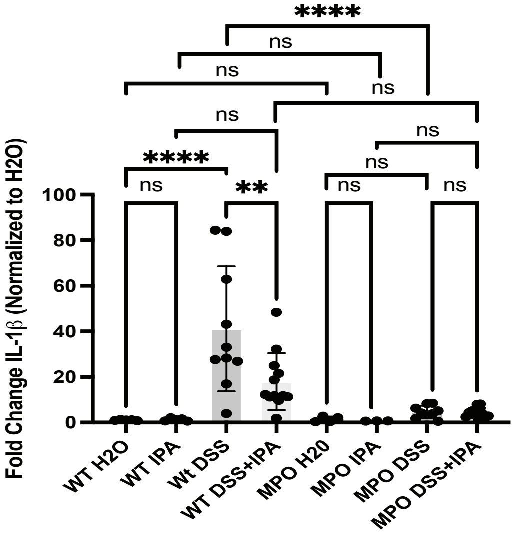

Fold Change IL-1β (Normalized to H2O)
0
20
40
60
80
100
WT H2O
WT IPA
Wt DSS
WT DSS+IPA
MPO H20
MPO IPA
MPO DSS
MPO DSS+IPA
****
ns
ns
ns
ns
ns
****
ns
**
ns
ns
ns

Supplemental 5: Analysis of 3-Cl-Tyr in Occludin. (A and B) EC-HPLC tracings of 1μM and 100nM standards of Tyr (~3min retention time) and 3-Cl-Tyr (~5min retention time). (C) Analysis of 3-Cl-Tyr in ZO-1 from T84 IEC exposed to PMN or activated MPO, n = 3 biological replicates. (D) Analysis of 3-Cl-Tyr in occludin, claudin-1, and JAM1 from water treated WT and MPO KO mice. Data is expressed as mean ± SD and the *p*-value determined by 1-Way ANOVA.

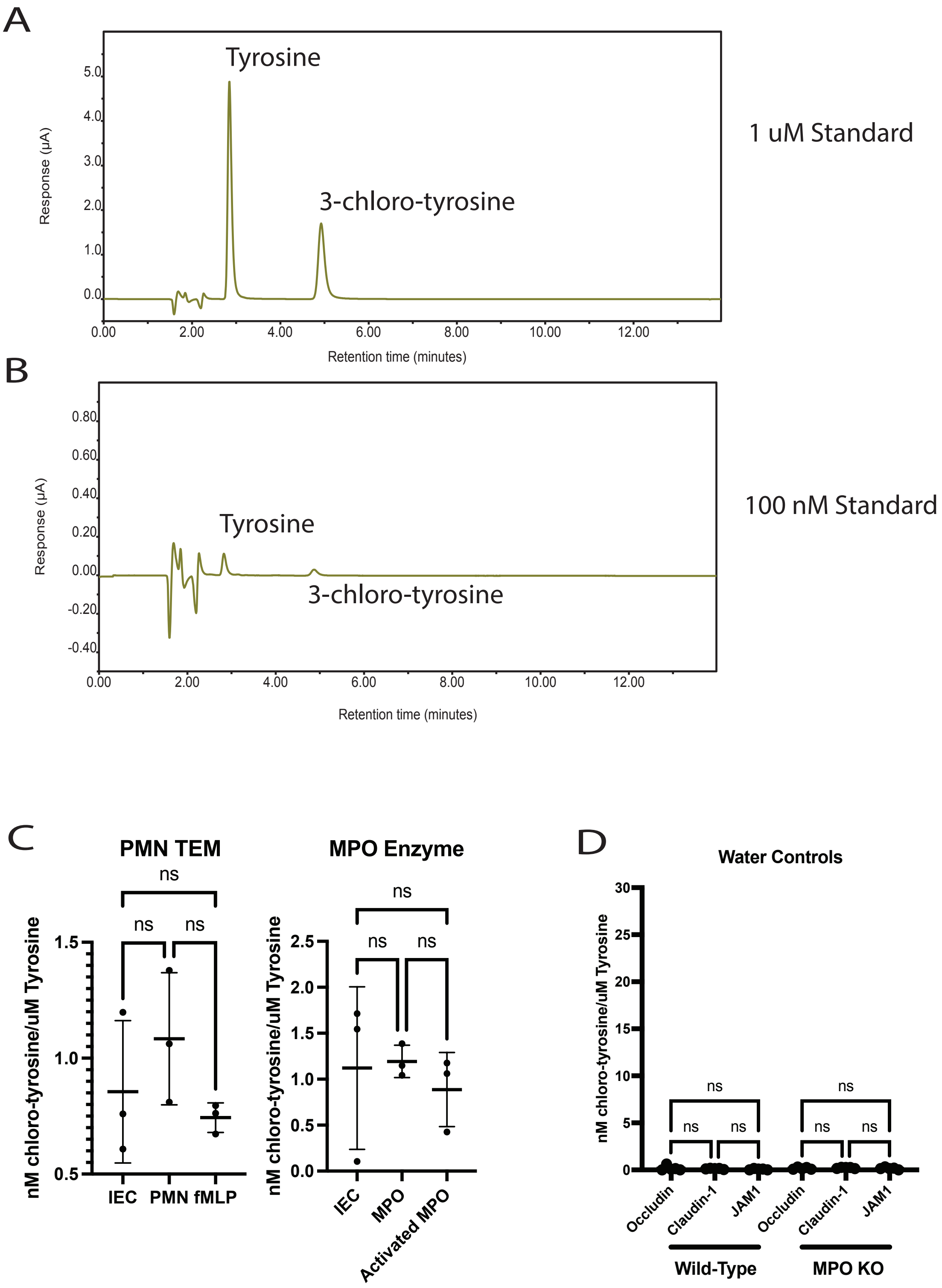
A
Tyrosine
3-chloro-tyrosine
Response (μA)
Retention time (minutes)
1 uM Standard
B
Tyrosine
3-chloro-tyrosine
Response (μA)
Retention time (minutes)
100 nM Standard
C
PMN TEM
ns
nM chloro-tyrosine/uM Tyrosine
IEC
PMN fMLP
MPO Enzyme
IEC
MPO
Activated MPO
D
Water Controls
nM chloro-tyrosine/uM Tyrosine
Occludin
Claudin-1
JAM1
Wild-Type
MPO KO

Supplemental 6: TJ rations of ZO-1 and occludin in T84 IEC. (A) Model of occludin peptide disrupting the TJ. (B) Analysis of ZO-1 TJ ratio in T84 IEC treated with 200 μg/ml scrambled, non-chlorinated, or chlorinated occludin peptide for 6hr. (C) Analysis of occludin TJ ratio in T84 IEC treated with 200 μg/ml scrambled, non-chlorinated, or chlorinated occludin peptide for 6hr. Arrows marks regions of decreased expression, non-linear staining, diffuse staining, or formation of puncta. Data includes individual measurements across 3 biological replicates. >30 total TJ measured across biological replicates, a total of 3 biological replicates (cells cultured at different time points) used. Data is expressed as mean ± SD and the *p*-value determined by 1-Way ANOVA. *** $p < 0.001$ and **** $p < 0.0001$.

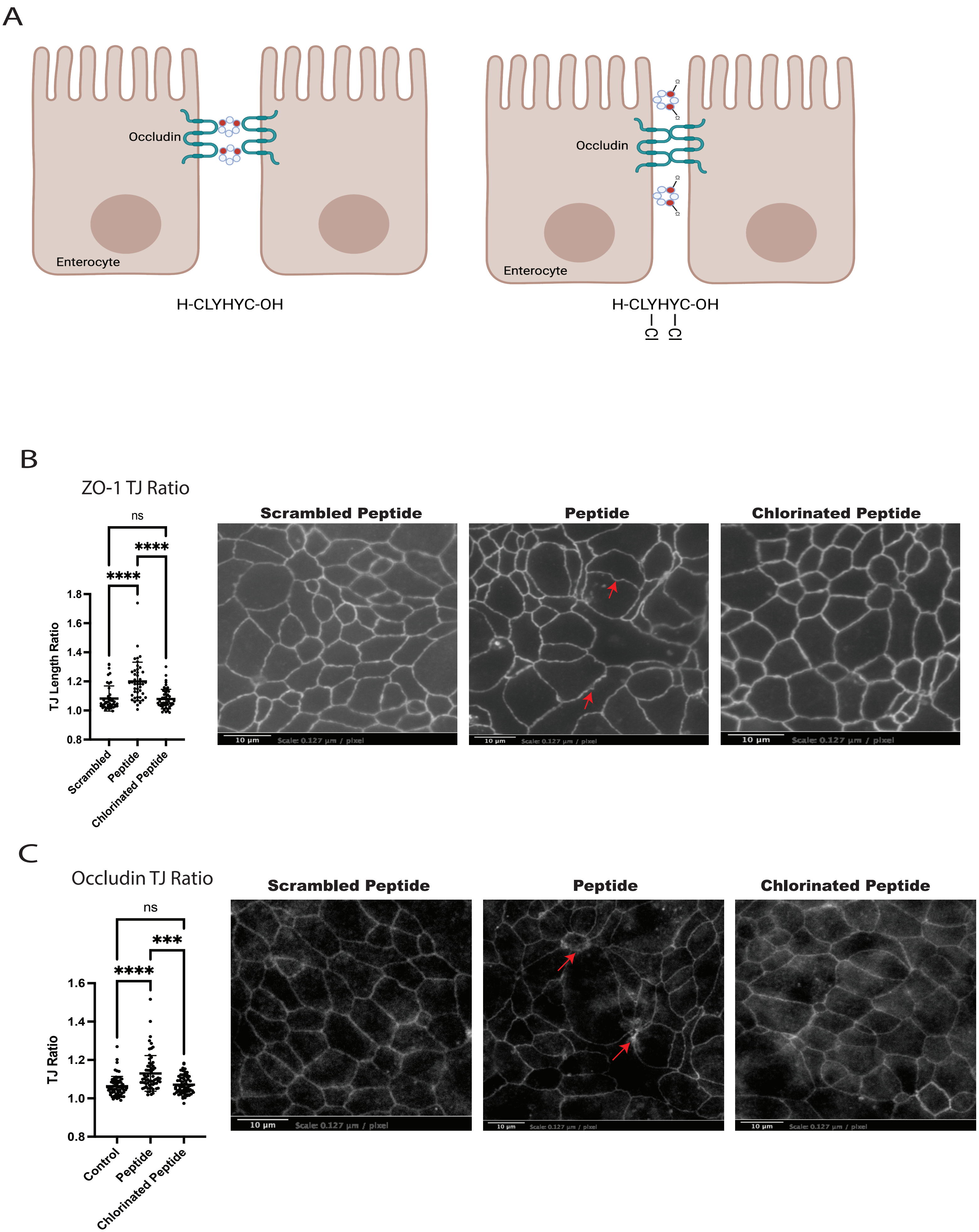
A
Occludin
Enterocyte
H-CLYHYC-OH
Occludin
Enterocyte
H-CLYHYC-OH
Cl Cl
B
ZO-1 TJ Ratio
ns
****
****
TJ Length Ratio
1.8
1.6
1.4
1.2
1.0
0.8
Scrambled
Peptide
Chlorinated Peptide
Scrambled Peptide
Peptide
Chlorinated Peptide
10 µm
Scale: 0.127 µm / pixel
C
Occludin TJ Ratio
ns
***
****
TJ Ratio
1.6
1.4
1.2
1.0
0.8
Control
Peptide
Chlorinated Peptide
Scrambled Peptide
Peptide
Chlorinated Peptide
10 µm
Scale: 0.127 µm / pixel

Supplemental 7: Occludin and ZO-1 staining in MPO treated IEC. (A) T84 IEC grown on 0.4 μm transwell inserts were grown to confluency and then treated with control, pH 5.0, 1 μg/ml MPO, 200 μM H_2O_2, or a combination of pH 5.0/MPO/H_2O_2 for 6hr. Following treatment the inserts were fixed in 1:1 methanol/acetic acid and stained for occludin. (B) Caco-2 IEC grown on 0.4 μm transwell inserts were grown to confluency and then treated with control, pH 5.0, 1 μg/ml MPO, 200 μM H_2O_2, or a combination of pH 5.0/MPO/H_2O_2 for 6hr. Following treatment the inserts were fixed in 1:1 methanol/acetic acid and stained for ZO-1. Arrows marks regions of mislocalization or decreased expression.

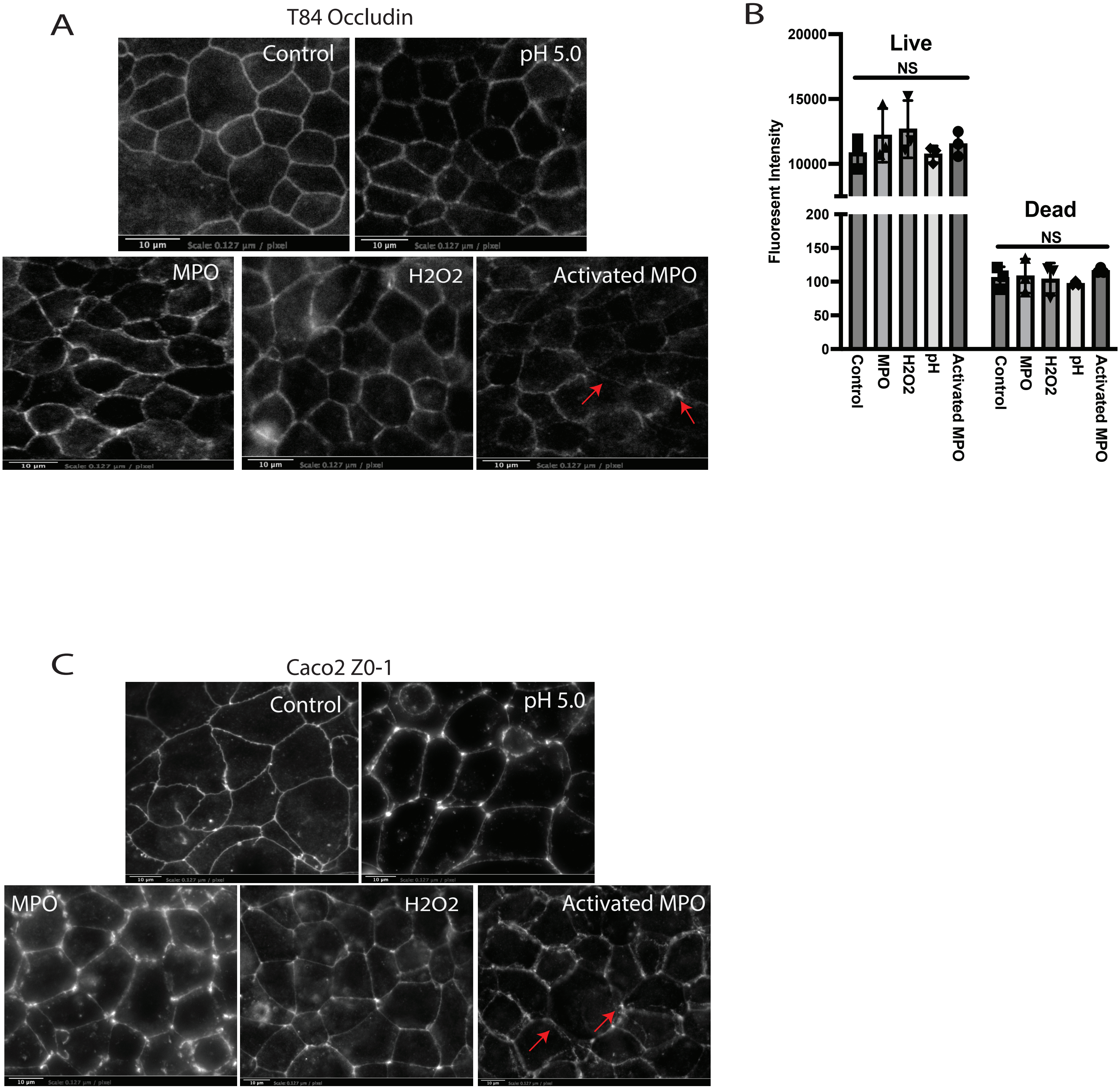

A
T84 Occludin
Control
pH 5.0
MPO
H2O2
Activated MPO
10 µm
Scale: 0.127 µm / pixel
B
Live
NS
Dead
NS
Fluoresent Intensity
20000
15000
10000
200
150
100
50
0
Control
MPO
H2O2
pH
Activated MPO
Control
MPO
H2O2
pH
Activated MPO
C
Caco2 Z0-1
Control
pH 5.0
MPO
H2O2
Activated MPO
10 µm
